# Multi-Modal Medical Image Fusion Based on FusionNet in YIQ Color Space

**DOI:** 10.3390/e22121423

**Published:** 2020-12-17

**Authors:** Kai Guo, Xiongfei Li, Hongrui Zang, Tiehu Fan

**Affiliations:** 1 Key Laboratory of Symbolic Computation and Knowledge Engineering of Ministry of Education, Jilin University, Changchun 130012, China; guokai15@mails.jlu.edu.cn (K.G.); xiongfei@jlu.edu.cn (X.L.); 2 College of Computer Science and Technology, Jilin University, Changchun 130012, China; 3 Information and Communication Company, State Grid Jilin Electric Power Co., Ltd., Changchun 130022, China; lihui20@mails.jlu.edu.cn; 4 College of Instrumentation and Electrical Engineering, Jilin University, Changchun 130012, China

**Keywords:** intuitive fuzzy processing, capture image details network, SeLU activation function, trace of a feature map, image entropy and cross entropy, YIQ color space

## Abstract

In order to obtain the physiological information and key features of source images to the maximum extent, improve the visual effect and clarity of the fused image, and reduce the computation, a multi-modal medical image fusion framework based on feature reuse is proposed. The framework consists of intuitive fuzzy processing (IFP), capture image details network (CIDN), fusion, and decoding. First, the membership function of the image is redefined to remove redundant features and obtain the image with complete features. Then, inspired by DenseNet, we proposed a new encoder to capture all the medical information features in the source image. In the fusion layer, we calculate the weight of each feature graph in the required fusion coefficient according to the trajectory of the feature graph. Finally, the filtered medical information is spliced and decoded to reproduce the required fusion image. In the encoding and image reconstruction networks, the mixed loss function of cross entropy and structural similarity is adopted to greatly reduce the information loss in image fusion. To assess performance, we conducted three sets of experiments on medical images of different grayscales and colors. Experimental results show that the proposed algorithm has advantages not only in detail and structure recognition but also in visual features and time complexity compared with other algorithms.

## 1. Introduction

Multi-modal medical image fusion is a combination of images of the same tissue or organ from multiple sensors and doctors can obtain relevant physiological information of the tissue or organ and its metabolic status from the fused image. Recently, the maturity of medical imaging technology provides various image information for medical diagnosis, including positron emission tomography (PET), computerized tomography (CT), single-photon emission computed tomography (SPECT), and magnetic resonance imaging (MRI) [1]. Medical images of various models provide rich, intuitive, qualitative, and quantitative physiological information of the human body to doctors and researchers from the perspective of vision and become an important technical means to diagnose various diseases. Due to the different imaging principles of different medical images, which reflect the anatomical or functional information of different tissues or organs, and have different sensitivity and resolution, they have their respective applicable scope and limitations. For example, CT images are sensitive to dense structures, such as bones or implants in the human body. MRI images are good at capturing soft tissue details and has anatomical information about organs. PET and SPECT images have the best effects in presenting organ metabolism and blood flow [2]. However, in clinical applications, single mode images often do not provide enough information for doctors and it is necessary to combine image information of different modalities. Multi-modal medical images are fused together to obtain more abundant information so as to understand the comprehensive situation of diseased tissues or organs and make accurate diagnosis or formulate appropriate treatment plans. Therefore, the multi-modal medical image fusion technology has been developing for a long time. In multi-modal medical image fusion, how to obtain useful information from multi-modal medical images and how to select the appropriate fusion method are still main issues.

In general, there are two main methods for multi-modal medical image fusion, namely spatial domain method and transform domain method. The spatial domain method is to select pixels [3] or regions [4] of the source image without transformation [5]. The transform domain method incorporates the corresponding transform coefficients, and the fused image can be obtained through inverse transform. In the transform domain method, there are various transforms, including discrete wavelet transform (DWT) [6], contourlet transform [7], dual tree complex wavelet transform (DTCWT) [8], curvelet transform (CVT) [9], and non-subsampled contourlet transform (NSCT) [10]. Most multi-scale transform (MST) fusion methods pay special attention to the structural information of the fused image, and some details are not well done. S. S. Paris et al. proposed an idea called local Laplacian filters (LLF) [11], which both ensures clear edges and enhances fused information. After that, doctors paid more attention to the biological metabolism information they were interested in, and the information of interest (IOI) algorithm came into being [12].

In the last five years, representation learning [13,14,15] become more and more popular in image fusion. Sparse representation (SR) was first introduced into image fusion by Li and Yang et al. [16]. With the development of sparse representation, sparse representation image fusion algorithms based on joint dictionary rise [17]. Then, low rank representation (LRR) based on dictionary learning [13] is proposed and applied to image fusion. SR method has high time complexity. It is usually divided into two steps. The first step is to build a dictionary through training, and the second step is to use a suitable fusion strategy for image fusion based on the constructed dictionary.

Recently, deep learning algorithms have been widely used in the field of images, with amazing results. Especially, the convolutional neural network (CNN) has brought new opportunities to the field of image fusion [18]. In [19], Song et al. used two convolutional neural networks to process space-time satellite images. Space-time satellite images include moderate-resolution imaging spectroradiometer (MODIS) and satellite images of different resolutions. Spatio-temporal satellite image fusion is the fusion of multi-resolution images. Specifically, two CNNs are used to perform super-resolution on Landsat images and extract image features, and then linear computing strategies are used to reconstruct medical images. In [20], Li et al. used the middle layer of the pre-trained visual geometry group (VGG) network to obtain the features of the image and then used the L1 norm and weighted average strategy to generate the details of the fused image. Prabhakar et al. [21] proposed a new multi-mode image fusion framework, which is composed of feature extraction layer, information fusion layer, and image reconstruction layer. The feature extraction layer adopts Siamese network architecture, and three layers of CNN constitute its reconstruction layer. Li et al. [22] came up with a Densefuse and they choose convolutional layers and dense block as encoding network in 2019.

As for deep learning fusion algorithm mentioned above, CNN-based fusion strategies only use results of the last layer as the image features, which will lose some useful information contained in the middle layer. Aiming at the uncertainty of multi-modal medical image affected by radiation and the loss of image information after fusion, we propose a new multi-modal medical image fusion framework. First, functional images are converted into YIQ color space to obtain its Y component. Next, the Y component of functional images and the corresponding structural images are convolved into DenseNet to obtain the fusion features. Then, the fusion features are input into the fusion network for feature fusion. Finally, we use the trained network and the previously obtained fusion features to reconstruct the fusion image.

For clarity, the main contributions of the paper can be described as follows:

- We preprocessed two images before fusion, reconstructed the non-membership function according to the relevant knowledge of fuzzy set theory, and obtained their membership hesitation images, which perfectly solved the uncertainty problem caused by the input images coming from different sensors.
- In view of the serious loss of image information in multi-mode medical image fusion, we proposed a new feature enhancement network inspired by DenseNet. At the same time, the gradient disappearance and model degradation are alleviated to some extent by using the new excitation function.
- In the fusion method, we use the trace of matrix to calculate the weight coefficient of each feature graph. The trace is the sum of eigenvalues of each characteristic graph. The eigenvalue is regarded as the importance value of different features in the matrix and can cover the fusion features in the most comprehensive way.
- As far as we know, it is the first time that the combined loss of sensible cross-entropy and structural similarity has been introduced in the training of a CNN-based multi-modal medical image fusion model. Cross entropy can better express the degree of retention of visual color information in fused images. However, the structure similarity is better in expressing edge and texture information in fusion images. Through introducing the combined loss of cross entropy and structural similarity, the trained multi-mode medical image fusion model has obvious advantages in both visual information retention and texture information acquisition.

The rest of the work is organized as follows. In Section 2, related work of the paper is described. In Section 3, the proposed multi-modal medical image fusion framework is presented in detail. The experimental results are given in Section 4, and conclusions and future work are presented in Section 5.

## 2. Related Work

### 2.1. Intuitionistic Fuzzy Sets

Intuitionistic fuzzy set is an improvement of the traditional fuzzy set [23,24,25]. The first generation of intuitionistic fuzzy sets introduced non-membership functions and the second-generation intuitionistic fuzzy sets introduced hesitancy functions between membership functions and non-membership functions, which makes the intuitionistic fuzzy set more complete. However, when dealing with ambiguity and uncertainty, intuitionistic fuzzy set is slightly better than the previous ones.

Atanassov [26] and Stoeva [27] proposed the first generation of intuitionistic fuzzy sets (IFS). Intuitionistic fuzzy set *F* in *X* can be symbolized with the essential condition.

(1) $$F=\{\left(x,\mu_{F}\left(x\right),\nu_{F}\left(x\right)\right)|x\in X\},$$

where the functions $\mu_{F}\left(x\right),\nu_{F}\left(x\right):X\in \left[0,1\right]$ represents the degree of membership and non-membership of an element *x* in *X*, respectively, with the essential condition $0\leq \mu_{F}\left(x\right)+\nu_{F}\left(x\right)\leq 1$.

Szmidt and Kacpryzk [28] introduced a new parameter $\pi_{F}\left(x\right)$ due to lack of knowledge when calculating the distance between fuzzy sets (FS), called hesitation. IFS is defined as follows based on the hesitation degree.

(2) $$F=\{\left(x,\mu_{F}\left(x\right),\nu_{F}\left(x\right),\pi_{F}\left(x\right)\right)|x\in X\},$$

where the condition $\mu_{F}\left(x\right)+\nu_{F}\left(x\right)+\pi_{F}\left(x\right)=1$ holds.

The research on intuitionistic fuzzy set theory has attracted great attention from scholars in relevant fields at home and abroad, and it has been applied to many fields, such as pattern recognition, data mining, information fusion, and information security.

### 2.2. DenseNet

**Figure 1:**
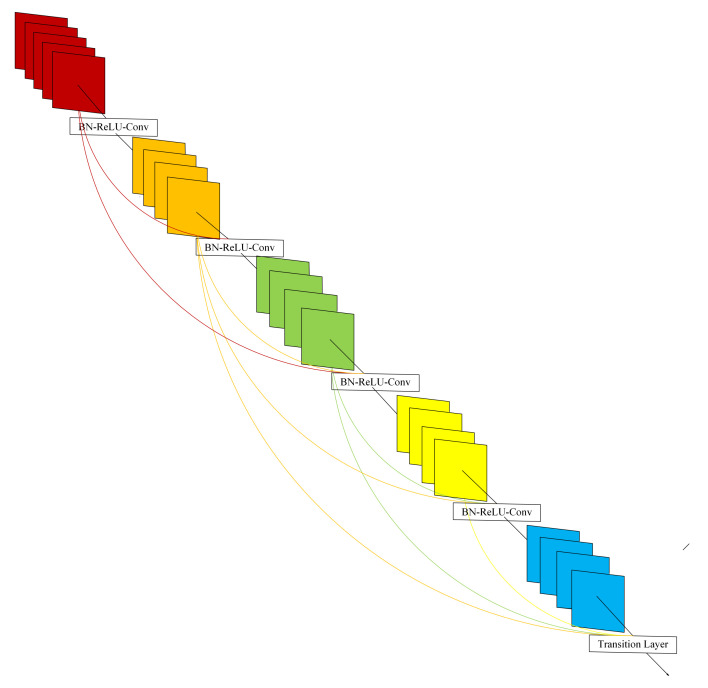
A 5-layer dense block with a growth rate of k = 4. Each layer takes all preceding feature-maps as input.

In this section, we briefly introduce DenseNet. Huang et al. [29] start with features and achieve better results and fewer parameters through the ultimate use of features. Under the premise of ensuring the maximum information transmission between layers in the network, all layers are directly connected. Figure 1 describes the layout of DenseNet. The input of the *l*th layer is the feature map output by each layer of the first l-1 layers.

(3) $$X_{l}=H_{l}\left(\left[x_{0},x_{1},\cdots x_{l-1}\right]\right),$$

where $\left[x_{0},x_{1},\cdots x_{l-1}\right]$ is the concatenation of all output feature maps of the first l-1 layer. Because of its dense connection, it is called dense convolutional network (DenseNet). To facilitate implementation, multiple inputs of $H_{l}\left(\cdot \right)$ are concatenated into a single tensor. Motivated by [30], $H_{l}\left(\cdot \right)$ is a composite function, which is consists of batch normalization (BN) [31], followed by a rectified linear unit (ReLU) [32] and a 3×3 convolution (Conv).

DenseNet architecture is suitable for image fusion because it has three advantages.

- This architecture can save as much information as possible in the process of image fusion.
- Due to the regularization effect of density connection, this model reduces the overfitting of experimental tasks.
- The model can improve the gradient of the network, making it easier to train.

### 2.3. YIQ Color Space

In recent years, the fusion of structural and functional images has led to important changes in the field of medical research, particularly in cancer diagnosis [1,33,34,35]. A functional image is usually considered as a color image. Combined with a structural image, it preserves more information about biological tissue than a single image. Functional images can be viewed as color images. Compared with single modality images, the fusion of functional images and structural images can provide more physiological information of tissues and organs. Color images are generally in RGB color space, which covers almost all colors that the human eye can distinguish. All three colors are treated equally because of their strong correlation. Once the composition of the RGB image changes, it is difficult to predict which colors will change. In multi-mode medical images, the channel numbers of functional images and structural images are different, so it is difficult to apply RGB color space. At the same time, if the coefficients of the R, G, or B components change due to strong correlation, the color of the fused image will also change. The details of the functional image and the color information should be separated from each other, so that the features of the structural image and the details of the functional image can be merged together, while the color information is easy to retain. In order to avoid the disadvantages of RGB color model, other color models, like IHS and YCrCb, have been introduced into the field of medical image fusion. Of course, they have their drawbacks [36,37]. In IHS color space, the three components cannot be completely independent of each other; in YCrCb color space, the blue and red offsets are not decomposed according to the color sensitivity of the human eye. The YIQ color space has great advantages in preserving color information. The color vision characteristics of the human eye indicate that the human eye has the strongest ability to distinguish between red and yellow, and the weakest ability to distinguish between blue and purple. There is a certain change, I corresponds to the chromaticity most sensitive to the human eye, and Q corresponds to the chromaticity least sensitive to the human eye. In this way, a narrower bandwidth can be used to transmit Q, and a wider frequency band can be used when an I signal is transmitted with a higher resolution. Corresponding to the digitization process, these components can be recorded with different numbers of bytes. These are advantages that color spaces, such as HSV, IHS, and CMY, do not have. At the same time, doctors rely heavily on color vision characteristics, and they need to use sensitive colors to judge whether the metabolism of organs or tissues is normal. Therefore, we, finally, chose the YIQ color space.

The conversion formula from RGB color space to YIQ color space is as follows [38]:

(4) $$\left[\begin{array}{c} Y\\ I\\ Q \end{array}\right]=\left[\begin{array}{ccc} 0.299 & 0.587 & 0.144\\ 0.596 & 0.274 & 0.322\\ 0.211 & 0.523 & 0.312 \end{array}\right]\times \left[\begin{array}{c} R\\ G\\ B \end{array}\right].$$

## 3. A New Framework for Image Fusion

**Figure 2:**
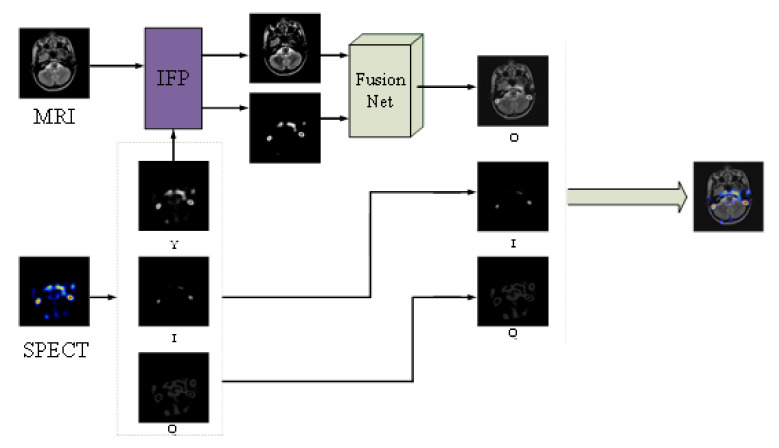
Our fusion framework.

In this section, our method will be introduced in detail. The framework of the method is shown in Figure 2.

**Figure 3:**
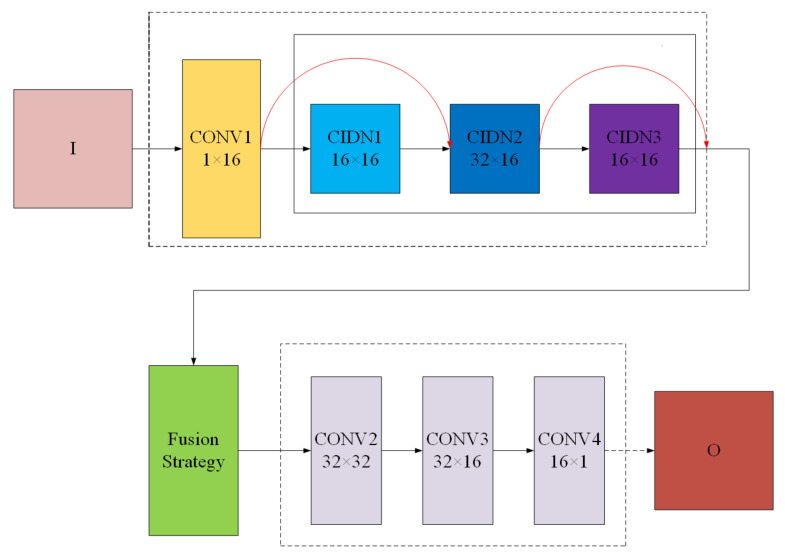
The details of FusionNet.

As shown in Figure 2, we select two registered multi-modal images, MRI and PET. First, the PET is decomposed into the YIQ color space to obtain three channels, and its gray channel Y is taken out, and the two images of Y and MRI are input to the intuitionistic fuzzy set processing module to remove some redundant features and enhance the salient features. Then, input the enhanced two images into FusionNet for image fusion. Finally, the O obtained after fusion is combined with the I and Q channels of PET, and the fusion image space is obtained by converting the YIQ color space to RGB color. In the whole framework, FusionNet, as the core part of the framework, eliminates the uncertain factors in the Y channel of functional images and structural images, extracts fusion features from CIDN, realizes feature fusion, and reconstructs the image after fusion. FusionNet is described in detail in Figure 3.

As we can see, FusionNet is composed of encoder, fusion strategy, and decoder. Encoder is composed of a convolutional layer and CIDN. CIDN contains three filters, among which CIDN2 plays an important role in feature multiplexing. In terms of fusion strategy, we choose the trace that can better show the matrix characteristics as the criterion for assigning weights in the strategy. The decoder is composed of three convolutional layers that are used to reconstruct the input image.

### 3.1. Intuitive Fuzzy Processing (IFP)

In an intuitionistic fuzzy set, the construction of membership degree, non-membership degree and hesitation function is the key step. The traditional fuzzy set processing mostly constructs the membership image by fixed functions, such as gaussian and trigonometric functions. These methods rely on prior knowledge to select appropriate functions for different kinds of images, and are difficult to be applied to complex multi-modal situations. Considering that entropy can reflect the amount of information in an image, what we care about in multi-modal medical image fusion is the retention degree of the image information after fusion. Considering that, if we only rely on the membership degree image and ignore the hesitation degree image, it is likely to lose some effective information of the medical image, we construct the non-membership function to obtain the non-membership image, and then we can calculate the image without missing the key information.

The following are the detailed steps to redefine the non-membership function of image *I*.

The image *I* of size and grayscale *L* is regarded as a set of units. Suppose *g* is each element of the image and $\nu_{F}\left(I\left(x,y\right)\right)$ is the degree to which element *g* does not belong to image set *I*.

(5) $$\nu_{F}\left(I\left(x,y\right)\right)=\frac{g_{\max}-g}{g_{\max}-g_{\min}},$$

where $g_{\min}$ and $g_{\max}$ represent the minimum and maximum values of image *I*. The corresponding non-membership image can be defined as

(6) $$\nu_{IFS}\left(I\left(x,y\right);\lambda \right)={\left[\nu_{F}\left(I\left(x,y\right)\right)\right]}^{\lambda }.$$

Our image to be fused can be expressed as

(7) $$\xi_{IFS}\left(I\left(x,y\right);\lambda \right)=1-\nu_{IFS}\left(I\left(x,y\right);\lambda .\right)$$

The value of parameter λ is determined by the selected image. Since a lot of IFSs can be obtained for an image by changing the value of λ, finding the optimal solution becomes the most important task, which needs to be realized by entropy. De Luca and Termini [39] proposed the definition of entropy in FS theory. Scholars in [28,40,41,42] have proposed different entropy measures based on IFS theory. Inspired by the above methods, we propose a new definition method of entropy for multi-modal medical images, and the definition formula is as follows:

(8) $$entory\left(IFS;\lambda \right)=\frac{1}{M\times N}\sum_{i=0}^{M-1}\sum_{j=0}^{N-1}\frac{2\mu_{IFS}\left(I(i,j);\lambda \right)\xi_{IFS}\left(I(i,j);\lambda \right)}{\mu_{IFS}^{2}\left(I(i,j);\lambda \right)+\xi_{IFS}^{2}\left(I(i,j);\lambda \right)}.$$

Through the above methods, we will obtain pre-processed multi-modal medical images to be fused.

### 3.2. FusionNet

FusionNet contains the convolutional layer and CIDN. The convolutional layer contains 3×3 filters to capture the rough features of medical images and CIDN is good at obtaining the detailed information of medical images. CIDN consists of three convolutional layers that also contain 3×3 filters. In our network, we use the second layer as the main feature reuse layer. Feature multiplexing layer takes the features of all previous layers as input, and then the output directly acts on the next layer and the fusion layer. This network structure is effective in multi-modal medical image fusion and reduces the loss of biological information in the fusion process. Our encoder can input images of any size, which is an advantage of our network as an encoder.

We chose a relatively novel fusion strategy in the fusion layer, which will be introduced in Section 3.2.3.

The decoder consists of three convolutional layers (3×3 filters). Its input comes from the output of the fusion layer, and we use this simple and effective architecture to reconstruct the fusion image.

#### 3.2.1. CIDN

Multi-modal medical image fusion focuses on the acquisition of image information from different sensors. However, it is not the better to get more feature information, which will result in blurring or even distortion of the final fusion image. The traditional DenseNet network has many feature multiplexing layers, which is not suitable for direct use in multi-modal medical image fusion. Inspired by DenseNet, we take the last three layers of the encoder as the characteristic multiplexing network and design the penultimate layer as the network multiplexing layer. It not only avoids the negative effect of repeated aggregation of many features in the middle layer on the final layer fusion effect but also solves the cost of feature redundancy in the time complexity of the algorithm.

CIDN will use SeLU as the activation function instead of the traditional ReLU. SeLU function is defined as follows:

(9) $$SeLU\left(x\right)=\lambda \left\{\begin{array}{c} x\;\mathrm{if}\;x>0\\ \alpha (e^{x}-1)\;\mathrm{if}\;x\leq 0 \end{array},\right.$$

where *x* is the input feature, and α is a constant greater than one.

As we all know, ReLU activation has many advantages. It can make the network training faster, while increasing the nonlinearity of the network. The most important thing is that it can prevent the gradient from disappearing and reduce overfitting. During the training process, some neurons “die”, that is, they stop producing anything but zero. In some cases, half of the neurons in the network will have the above situation, especially when high learning rates are used. Once the neuron’s weight is updated during training so that its input weighted sum is negative, it starts to output zero. The reason is that, when the input is negative, the gradient of the ReLU function is zero, and the neuron can only output zero.

In view of the above problems with ReLU activation function, we use SeLU as the activation function of CIDN. First, the SeLU activation function can accelerate the convergence speed of the network because the internal normalization speed is higher than the external normalization speed. Second, it avoids the “ReLU dead zone” problem. Finally, when the input is greater than 0, activating the output amplifies the input. This will greatly improve the efficiency of CIDN in processing multi-modal medical images.

#### 3.2.2. Loss Function

In the training stage, we temporarily ignore the fusion layer and select the existing image data set to try to train our encoder and decoder network to reconstruct the input image. After the weights of encoder and decoder are determined, the appropriate fusion strategy is adopted to fuse the depth characteristics obtained by encoder. The biggest advantage of this method is that it can design an appropriate fusion method according to the characteristics of the source image, which will lay a solid foundation for adaptive fusion in the future.

In order to obtain a better reconstructed image, we made great efforts to select the loss function. According to the characteristics of functional images and structural images, we intend to use the cross entropy loss function and structural similarity loss function to form the mixed loss function. We choose the mixed loss function to train the encoder and decoder. The mixed loss function is shown as follows:

(10) $$Loss_{mix}=\alpha Loss_{cross\_entropy}+\beta Loss_{ssim},$$

where alpha and beta are the weights of the two loss functions. Given the different characteristics of different organs and tissues, some organs are more concerned with functional information, while others have more important structural information. Therefore, it is necessary to use two parameters to adjust the loss function in the reconstruction of the fused image neural network.

The cross entropy loss function is described as

(11) $$Loss_{cross\_entropy}=-\sum I\log(O)+(1-I)\log(1-O).$$

The structural similarity loss function is described as

(12) $$Loss_{ssim}=1-SSIM\left(I,O\right),$$

where *I* represents the input image, and *O* represents the output image. *SSIM*(.) represents the structural similarity operation, and structural similarity will be described in detail in the section of evaluation metrics.

**Figure 4:**
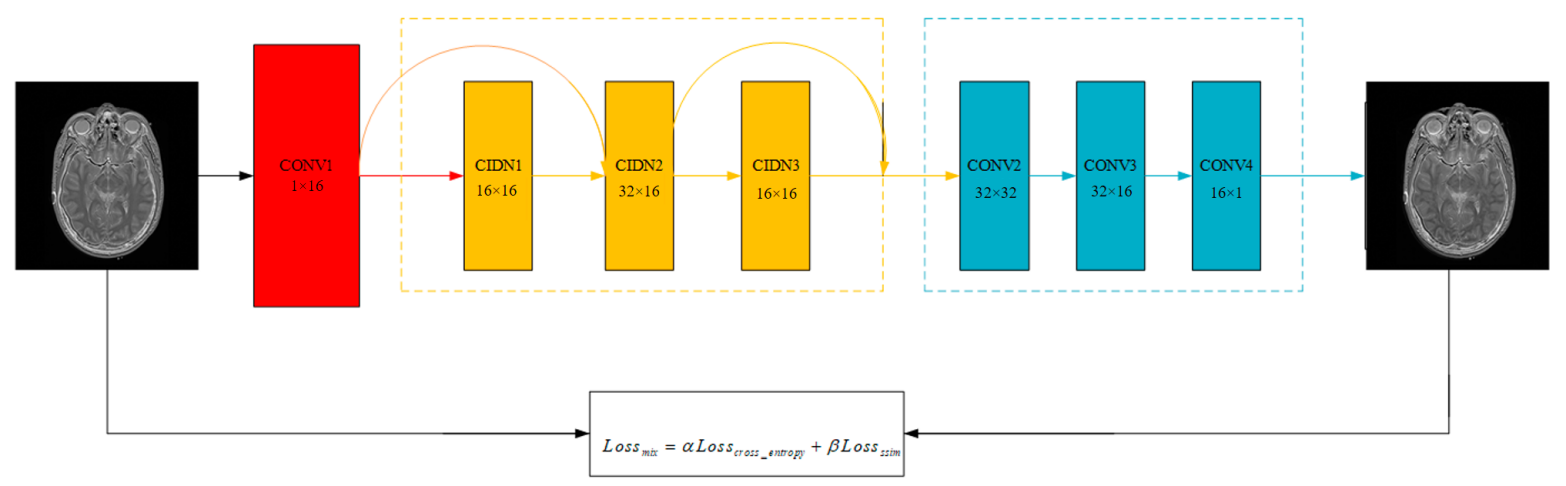
The evolution of images through encoder and decoder.

As shown in Figure 4, our images reduce a lot of information loss when passing through the encoder and decoder which use this loss function.

#### 3.2.3. Fusion Strategy

There are many ways to fuse the convolution function of multiple inputs. The two most representative methods are addition strategy and l1-norm strategy. The performance of the addition strategy has been fully demonstrated in [22], but, for the fusion of salient features, this method is particularly rough. As for l1-norm strategy, it has a large amount of computation, high time complexity, and too much redundant information. In view of the above problems in the fusion strategy, the trace of the feature graph is the sum of all the eigenvalues of the matrix of the feature graph. The trace of the matrix is described as

(13) $$Tr\left(B\right)=\sum_{i=1}^{n}b_{ii}.$$

*B* is the matrix and $b_{ii}$ is the diagonal member of the matrix *B*. We will calculate the weight value of feature maps according to their traces.

(14) $$\omega_{i}\left(x,y\right)=\frac{\sum_{1}^{n}Tr\left(\varphi_{i}^{1:n}\left(x,y\right)\right)}{\sum_{i=1}^{k}\sum_{1}^{n}Tr\left(\varphi_{i}^{1:n}\left(x,y\right)\right),}$$

where $\varphi_{i}^{1:n}\left(x,y\right)$ indicates the feature maps, and we calculate $f^{n}\left(x,y\right)$ that represents the fused feature maps by

(15) $$f^{n}\left(x,y\right)=\sum_{i=1}^{k}\omega_{i}\left(x,y\right)\times \phi_{i}^{n}\left(x,y\right),$$

where *k* is the index of feature maps which are obtained from input images. The fused features will be concatenated and input into decoder. Finally, our fused image *F* is obtained through image reconstruction.

## 4. Experimental Results and Discussion

### 4.1. Experimental Settings

In this section, we first used the image data set to train the whole network for feature extraction and image reconstruction ability. Then, we performed three fusion experiments of different modal medical images. In the process of experimental analysis, subjective expert scores and objective fusion metrics are used. As for the expert score, we drew the obtained score into a histogram using the mean value to facilitate comparison and ten metrics were taken to evaluate the fusion results. The best metrics have been marked in bold.

#### 4.1.1. Data Set and Compared Algorithms

The images to be merged in the experiment were taken from the Harvard Brain Database. Each group of images is composed of functional images and structural images that have been registered. We choose MRI as the structural image of the source image. MRI images have a flow blank effect that allows blood vessels and soft tissue to be easily separated. For the corresponding functional image, we selected several different functional images. The features of each functional image are described in the corresponding part of the experiment.

In the selection of comparison algorithm, we adopt several representative methods. Among them, there are pyramidal wavelet transform, such as DTCWT and NSCT [43]. A sparse representation image fusion algorithm called Laplacian pyramid sparse representation (LPSR) [44] (download from: http://home.ustc.edu.cn/~liuyu1/) is also in our contrast algorithm. There are also popular deep learning image fusion algorithms, such as Fusion convolutional neural network based algorithm (FusionCNN) [45] and dual-discriminator conditional generative adversarial network based algorithm (DDcGAN) [46]; besides, guided filtering fusion algorithm (GFF) [47] (download from: http://xudongkang.weebly.com/) and internal generative mechanism (IGM) [48] are also indispensable two contrast algorithms. The code of all the contrast algorithms comes from the relevant papers and some from the relevant academic forums. The parameters are the default settings.

#### 4.1.2. Training Settings and Fusion Metrics

Microsoft Common Objects in COntext (MS-COCO) data set was selected as the data set for training FusionNet feature extraction and reconstruction ability. As is well known, MS-COCO data set is a large, rich image data set. The data set is targeted at scene understanding. It is mainly intercepted from complex daily scenes, and the targets in the image are demarcated by precise segmentation. It is appropriate to use this data set to train the ability of network image reconstruction. We selected 80,000 images from MS-COCO data set [49], adjusted the size of these images to 256×256, and converted them to grayscale images, using them to train our network. Learning rate, batch size, epochs, and parameter α are set as 0.0005, 32, 10, and 500, respectively. Our training was realized with NVIDIA RTX 2080 GPU and Tensorflow is utilized as the back end for the network architecture.

There are generally many evaluation indicators for image fusion. We selected five types of objective evaluation indicators in this article. They are based on statistical characteristics, amount of information, structural similarity, visual fidelity, and Piella model. In terms of statistical characteristics, we choose to be good at describing the average gradient (AG) of image sharpness and the root mean square error (RMSE) of captured image differences; in terms of information, in addition to mature information entropy (EN) and cross-entropy (CE) indicators, we use feature mutual information (FMI) [50] to improve image information and the lack of quantitative assessment. Structural similarity (SSIM) [51] and Piella model [52] are complementary in the evaluation of image structure. Because of the important structural information in our fused image, it is obtained from MRI images. Since the other source image is a functional image, we additionally chose visual fidelity (VIF) [53] as the last evaluation metric of the fused image.

In the field of image fusion, mutual information is used to represent the similarity of image intensity distribution between the fused image and the source image. Traditional mutual information calculation is based on pixel points, but the process of image fusion is a process of feature retention. Therefore, we should pay more attention to feature mutual information, which is more suitable for measuring the quality of fused images. Feature mutual information uses "gradients", "DCT", and "wavelets" to find out whether edge and contour information exists in the fused image. Feature mutual information is defined as follows:

(16) $$FMI=\sum_{f,a}P_{FA}\left(i,j,k,l\right)\log\frac{P_{FA}\left(i,j,k,l\right)}{P_{F}\left(i,j\right)P_{A}\left(k,l\right)}+\sum_{f,b}P_{FB}\left(i,j,k,l\right)\log\frac{P_{FB}\left(i,j,k,l\right)}{P_{F}\left(i,j\right)P_{B}\left(k,l\right)},$$

where $P_{FA}$ and $P_{FB}$ are the joint distribution between the fused image F and each of the source images A or B.

Average gradient can be used to measure the sharpness of the image to analyze the detail and texture of the fused image. The larger the average gradient value is, the richer the retained information of the fused image will be and the better the fusion effect will be. In addition, *AG* is an evaluation metrics independent of standard reference images and suitable for medical image fusion.

(17) $$AG=\frac{1}{M\times N}\sum_{i=1}^{M}\sum_{j=1}^{N}\sqrt{\frac{1}{2}\left({\left(F(i,j)-F(i+1,j)\right)}^{2}+{\left(F(i,j)-F(i,j+1)\right)}^{2}\right)}.$$

The root mean square error is a special measure of the fusion accuracy of the fused image and the source image. Assuming that the size of the image is M×N, F(i,j) represents the pixel in which the position is (i,j) in the fused image. $\bar{F}\left(i,j\right)$ represents the pixel in which the position is (i,j) in the source image. The mean square error can be described as

(18) $$RMSE_{sf}=\sqrt{\frac{1}{M\times N}\sum_{i=1}^{M}\sum_{j=1}^{N}{\left(F\left(i,j\right)-\bar{F}\left(i,j\right)\right)}^{2}.}$$

The root mean square error of multimodal medical image fusion is defined as follows:

(19) $$RMSE=\omega_{a}RMSE_{af}+\omega_{b}RMSE_{bf},$$

where *a* and *b* are the source images, and *f* is the fusion image. $\omega_{a}$ and $\omega_{b}$ are usually set to 0.5.

As a quality metric, $Q_{ab}^{f}$ plays an important role in image fusion. It is defined as follows:

(20) $$Q_{ab}^{f}=\frac{1}{\left|\omega \right|}\sum_{\omega \in W}\left(\lambda \left(\omega \right)Q_{0}\left(a,f\left|\omega \right.\right)+\left(1-\lambda \left(\omega \right)\right)Q_{0}\left(b,f\left|\omega \right.\right)\right),$$

(21) $$Q_{0}\left(a,b\right)=\frac{1}{\left|W\right|}\sum_{\omega \in W}Q_{0}\left(a,b\left|\omega \right.\right),$$

where *W* is the family of all windows, and $\left|W\right|$ is the cardinality of *W*. Starting from the top-left corner of the two images *a*, *b*, a sliding window of fixed size (with *n* pixels) moves pixel by pixel over the entire image until the bottom-right corner is reached. For each window ω, the local quality metric $Q_{0}\left(a,b|\omega \right)$ is computed for the values a(i,j) and b(i,j) where pixels (i,j) lie in the sliding window ω. Thus, in regions where image *a* has a large saliency compared to *b*, the quality metric and (a,b,f) are mainly determined by the input image *a*. On the other hand, in regions where the saliency of *b* is much larger than that of *a*, the metric $Q_{0}\left(a,b|\omega \right)$ is determined mostly by input image *b*.

Structural similarity index measure: *SSIM* is the widely used metric which models the loss and distortion between two images according to their similarities in light, contrast, and structure information. Mathematically, *SSIM* between images *x* and *y* can be defined as follows:

(22) $$SSIM_{xy}=\sum_{x_{i},y_{i}}\frac{2\mu_{x_{i}}\mu_{y_{i}}+c_{1}}{{\mu_{x_{i}}}^{2}+{\mu_{y_{i}}}^{2}+c_{1}}\cdot \frac{2\sigma_{x_{i}}\sigma_{y_{i}}+c_{2}}{{\sigma_{x_{i}}}^{2}+{\sigma_{y_{i}}}^{2}+c_{2}}\cdot \frac{{\sigma_{x_{i}}}_{y_{i}}+c_{3}}{\sigma_{x_{i}}\sigma_{y_{i}}+c_{3}}.$$

$Q_{w}$ gives an indication of how much of the salient information contained in each of the input images has been transferred into the fused image without introducing distortions. It is a different kind of fusion quality metrics, by giving more weight to those windows in which the input images are more significant. These areas are likely to be perceptually important parts of an undulating landscape. Therefore, when determining the comprehensive quality metric, the fusion image quality of these areas is particularly important. The overall saliency of a window is defined as $C\left(\omega \right)=\max\left(s\left(a\left|\omega \right.\right),s\left(b\left|\omega \right.\right)\right)$. The weighted fusion quality metric is then defined as

(23) $$Q_{w}\left(a,b,f\right)=\sum_{\omega \in W}c\left(\omega \right)\left(\lambda \left(\omega \right)Q_{0}\left(a,f|\omega \right)\right)+\left(1-\lambda (\omega )\right)Q_{0}\left(b,f|\omega \right).$$

$Q_{e}$ considers some aspect of the HVS, namely the importance of edge information. Note that we can evaluate $Q_{w}$ above using ’edge images’ (e.g., the Euclidean norm of the horizontal and vertical gradient images) instead of the original grey-scale images *a*, *b*, and *f*. Let us denote the edge image corresponding with *a* by $a^{\prime }$. Now, we combine $Q_{w}\left(a,b,f\right)$ and $Q_{w}\left(a^{\prime },b^{\prime },f^{\prime }\right)$ into a so-called edge-dependent fusion quality metric by

(24) $$Q_{e}\left(a,b,f\right)=Q_{w}{\left(a,b,f\right)}^{1-\alpha }\cdot Q_{w}{\left(a^{\prime },b^{\prime },f^{\prime }\right)}^{\alpha },$$

where the parameter α∈[0,1] expresses the contribution of the edge images compared to the original images: the closer α is to 1, the more important is the edge image.

Image entropy is a statistical form of image features, which reflects the average amount of information in the image. When we do image quality assessment, we generally use the image’s two-dimensional entropy. Compared with the one-dimensional entropy of the image, the two-dimensional entropy of the image not only represents the information contained in the aggregation features of the image grayscale distribution but also adds the grayscale characteristic information. The image entropy formula can be described as

(25) $$EN=-\sum_{x=0}^{M-1}\sum_{y=0}^{N-1}\frac{f(x,y)}{M\times N}\log\frac{f(x,y)}{M\times N},$$

where (*x*,*y*) represents the position of the pixel in the image, and *f*(*x*,*y*) represents the pixel value at (*x*,*y*). M×N represents the size of the image.

The image cross entropy is expressed as follows:

(26) $$D\left(P:Q\right)=\sum_{i=1}^{M}\sum_{j=1}^{N}p_{ij}\log\frac{p_{ij}}{q_{ij}}+\sum_{i=1}^{M}\sum_{j=1}^{N}q_{ij}\log\frac{q_{ij}}{p_{ij}}.$$

In the scene of image fusion, if *P* is the probability distribution of source image, *Q* is merged with the source image size in the image of the probability distribution of local image; depending on the image of cross entropy, the definition of the consistent *P* and *Q*, the cross entropy value is smaller, says the template image, and the greater the similarity between the local image in real time.

The cross entropy of multimodal medical image fusion is described as

(27) $$CE=\eta_{a}D\left(a:f\right)+\eta_{b}D\left(b:f\right),$$

where *a* is the MRI image, *b* is the PET image, and *f* is the fusion image. In a survey of fifty physicians in the department of Neurology, thirty-five of them were more interested in the structural information in the fused images, while the rest were more interested in the color information in the fused images. So, $\eta_{a}$ is 0.7, and $\eta_{b}$ is 0.3.

Visual information fidelity (VIF) is a measure of information fidelity which is consistent with the human visual system. The process of obtaining this index value is complex. First, filter and divide the source images and the fused image into different blocks. Next, evaluate the visual information of each block. Then, calculate the VIF value of each subband. Finally, calculate the overall measurement. The larger VIF indicates that the fusion method has good performance.

#### 4.1.3. Subjective Evaluation Methods

**Figure 5:**
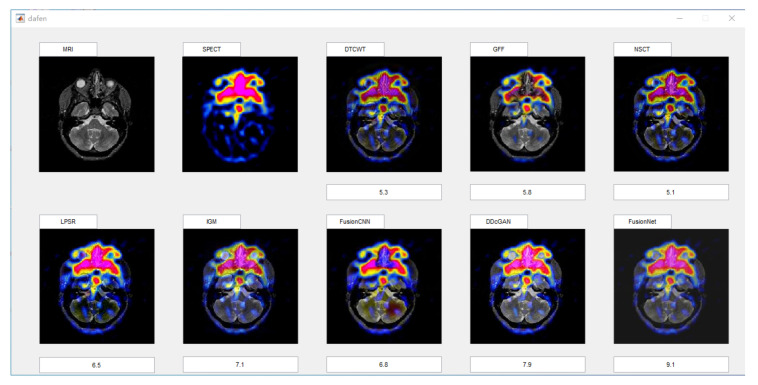
Interface of subjective scoring system.

Subjective evaluation methods generally rely on doctors in the field of organizing medical imaging to evaluate the visual effects of fused images. The evaluation method is relatively reliable. After all, the evaluation results are based on the doctors’ years of experience. However, there are also differences in the scores caused by the difference between the field of personal expertise and the research direction. The objective method predicts the visual quality of the fused image by modeling the human visual system, which can avoid the disadvantages of the subjective method. However, due to the complexity of the human visual system, modeling is impossible, so the evaluation result will deviate from human judgment. In our experiment, we used the above two methods to compare our algorithm with another seven representative algorithms. In order to minimize the interference of other factors on the subjective evaluation, we selected 10 male doctors and 10 female doctors in different hospitals, all of whom were from the medical imaging department. In order to reduce the impact of the environment on them, the assessment work is carried out in the same office. All images will be displayed on the computer monitor at the same resolution, so that you can ensure that everyone sees the same quality fused image. Scoring is done on a MATLAB GUI, which provides an enlarged tool for doctors to check details. The GUI is shown in Figure 5.

Doctors could give a score between 1 and 10 based on the texture, detail, and color changes in the fused image. For each fusion image, we will calculate its average score and variance as its subjective score. In view of the fact that there are three types of our functional images in the experiment, in the corresponding three types of fused images, we will select four groups of representative fused images for subjective scoring for each type.

#### 4.1.4. Parameters Selection

In this part, we focus on the training details of the encoder and decoder in FusionNet. First, the data set we use is 80,000 images from the MS-COCO data set. In learning rate, epoch, and batch size, since the value of batch size does not affect the calculation time, it is limited by hardware memory. According to Leslie’s theory, we set the batch size to 32 according to the actual situation of our hardware memory. Learning rate determines whether the objective function can converge to the local minimum and when it converges to the minimum. A proper learning rate can make the objective function converge to a local minimum in a proper time. So, we have to get an appropriate learning rate through experiments. Therefore, whether the setting of the learning rate is appropriate has a great impact on the performance of the model. The learning rate is generally set to a large number at the beginning; the purpose is to learn fast. Later, the model training was unstable. So, after a certain number of rounds, the learning rate should be gradually reduced. At this time, the convergence speed is slow, and it is easy to overfit. So, we use exponential decay learning rate. The formula is as follows:

(28) $$lr=0.95^{epoch\_num}\cdot lr_{0}.$$

lr represents the learning rate after decay, $lr_{0}$ represents the learning rate before decay, and epoch_num represents the number of iterations.

Since epoch should be greater than 1, and for our data set composed of 80,000 pictures, the value of epoch is related to whether our model is under-fitting or over-fitting. In order to eliminate the interference of human factors, we randomly generated one hundred sets of learning rates and epochs, and then decayed them exponentially. Finally, according to whether the model converges too slowly and cannot be learned, or converges too fast and loses a lot, decide which group is the best solution in the end. After comparing one by one, we selected the set of parameters with a learning rate of 0.0005 and an epoch of 10. Our model can obtain the optimal space under this parameter.

**Figure 6:**
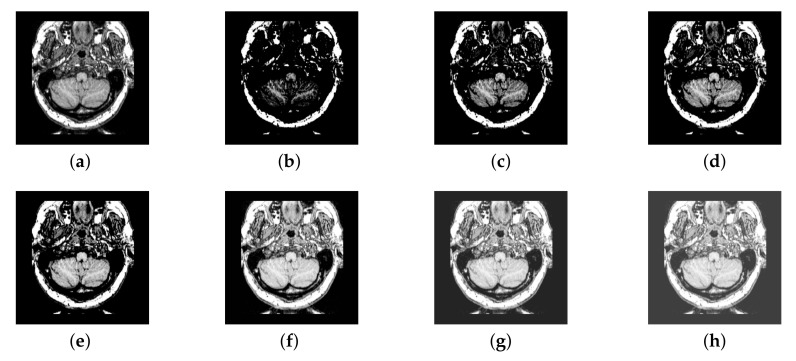
Source image and its image reconstructed by different algorithms): (**a**) Source image, (**b**) λ = 200, (**c**) λ = 300, (**d**) λ = 400, (**e**) λ = 500, (**f**) λ = 600, and (**g**) λ = 700, (**h**) λ = 800.

In previous intuitionistic fuzzy sets, scholars usually set λ to the order of 10 squares. Here, we set λ to 200, 300, 400, 500, 600, 700, and 800, respectively. Then, the image enhancement experiment is carried out, and the experimental results are shown in Figure 6. According to the results, we can find that, when λ is set to 500, the result is better than others.

### 4.2. The Fusion of MRI-SPECT

SPECT image can absorb radionuclide distribution diagram from different directions in vivo and draw the distribution. Three-dimensional reconstruction diagram of radionuclides in each cross section in vivo after computer comprehensive processing. It is something that structural MRI does not have. So, the combination of the two could allow doctors to get more accurate physiological information.

**Figure 7:**
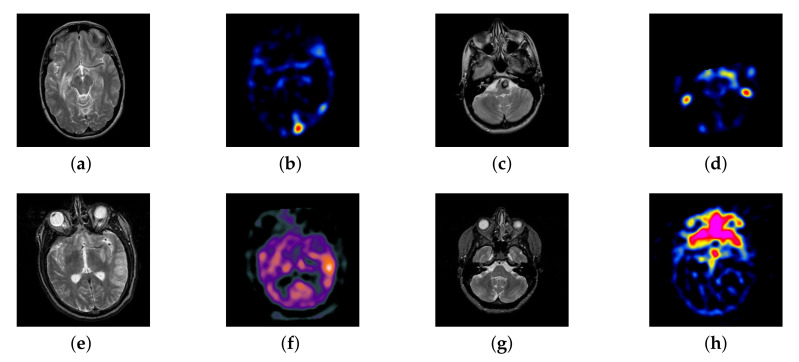
Four pairs of magnetic resonance imaging (MRI)-single-photon emission computed tomography (SPECT) source images: (**a**,**c**,**e**,**g**) are MRI images; (**b**,**d**,**f**,**h**) are SPECT images.

**Figure 8:**
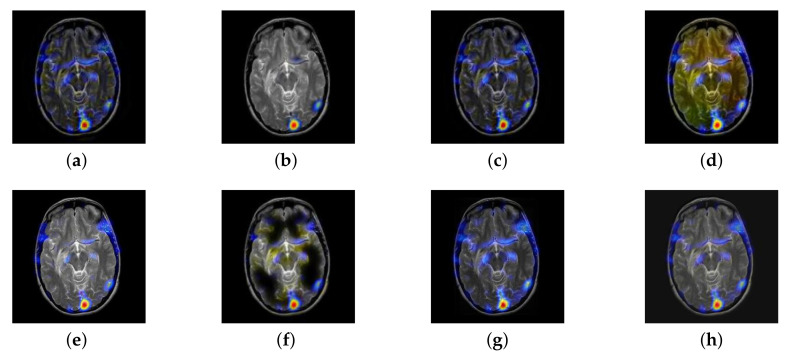
Fused medical images obtained by different algorithms (Figure 7a,b): (**a**) dual tree complex wavelet transform (DTCWT), (**b**) guided filtering fusion (GFF), (**c**) non-subsampled contourlet transform (NSCT), (**d**) Laplacian pyramid sparse representation (LPSR), (**e**) internal generative mechanism (IGM), (**f**) Fusion convolutional neural network based (FusionCNN), (**g**) dual-discriminator conditional generative adversarial network based (DDcGAN), and (**h**) FusionNet.

**Figure 9:**
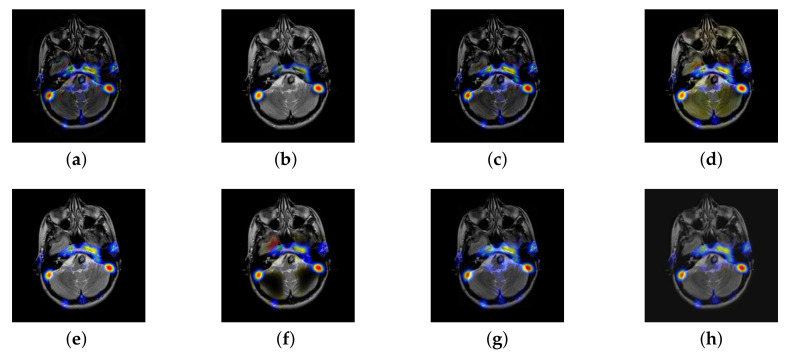
Fused medical images obtained by different algorithms (Figure 7c,d): (**a**) DTCWT, (**b**) GFF, (**c**) NSCT, (**d**) LPSR, (**e**) IGM, (**f**) FusionCNN, (**g**) DDcGAN, and (**h**) FusionNet.

**Figure 10:**
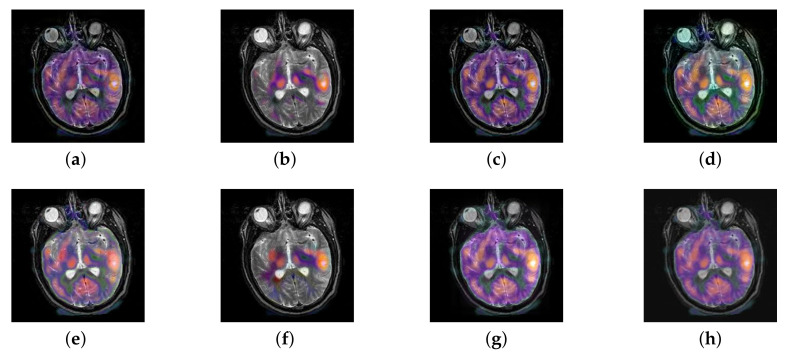
Fused medical images obtained by different algorithms (Figure 7e,f): (**a**) DTCWT, (**b**) GFF, (**c**) NSCT, (**d**) LPSR, (**e**) IGM, (**f**) FusionCNN, (**g**) DDcGAN, and (**h**) FusionNet.

**Figure 11:**
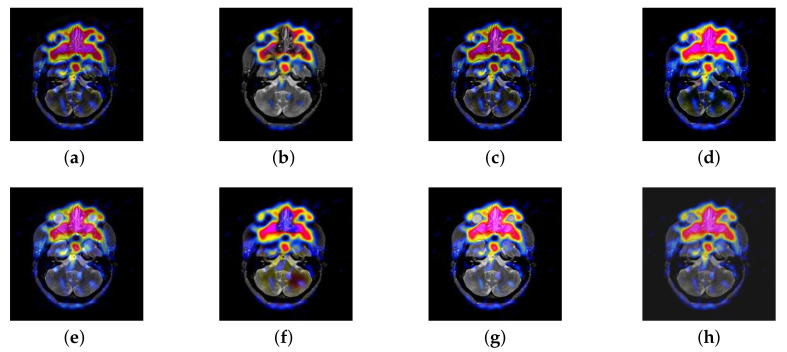
Fused medical images obtained by different algorithms (Figure 7g,h): (**a**) DTCWT, (**b**) GFF, (**c**) NSCT, (**d**) LPSR, (**e**) IGM, (**f**) FusionCNN, (**g**) DDcGAN, and (**h**) FusionNet.

In this section, there are four multi-modal image sets and each set is consist of MRI image and SPECT image that are corresponding to the sanme location slice of the brain as shown in Figure 7. Among them, Figure 7e,f are captured from patients who have suffered a subacute stroke. Figure 7a–d,g,h are captured from patients who have brain tumor. The fused images with different fusion methods based on DTCWT, NSCT, GFF, LPSR, IGM, DDcGAN, FusionCNN, and the proposed methods are shown in Figure 8, Figure 9, Figure 10 and Figure 11. It can be seen that the fused images obtained by LPSR and FusionCNN algorithm have serious color distortion. Based on DTCWT and NSCT algorithm, the fusion image structure information is not obvious. The fused images obtained by GFF and IGM algorithm contain almost no color information, which is not conducive for doctors to make correct diagnosis. The image obtained by the DDcGAN algorithm saves the color information in the SPECT to a great extent; however, the brightness of the fused image is too large, which causes the image to have no sense of hierarchy and the contrast to decrease. By comparing with other algorithms, we find that our algorithm has good color retention effect, clear structure information, moderate brightness, and no artifacts.

**Figure 12:**
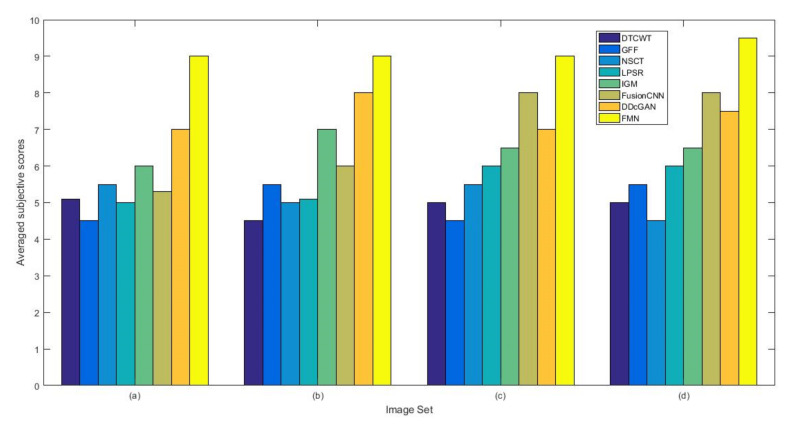
The averaged subjective scores of the fused images (MRI-SPECT): (**a**) is composed of eight images in Figure 8, (**b**) is composed of eight images in Figure 9, (**c**) is composed of eight images in Figure 10, (**d**) is composed of eight images in Figure 11.

**Figure 13:**
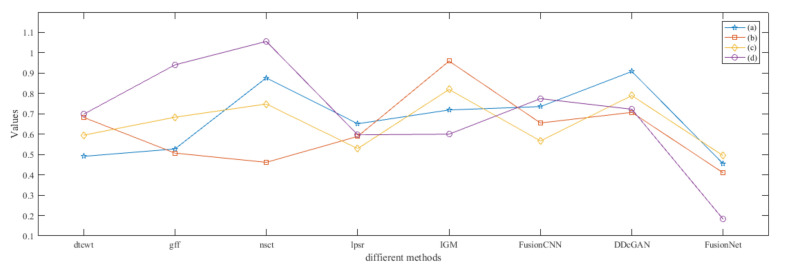
The standard deviations of subjective scores of the fused images (MRI-SPECT).

**Figure 14:**
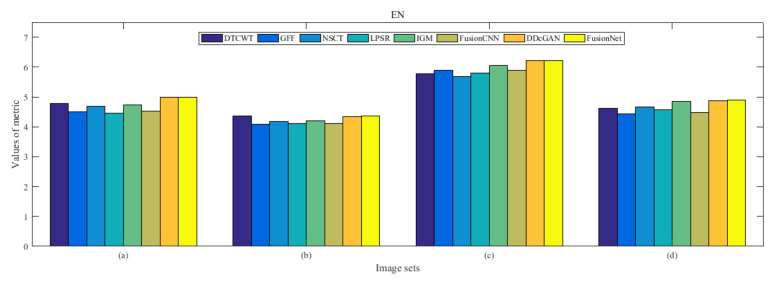
Values of entropy (EN) in the fused images (MRI-SPECT): (**a**) is composed of eight images in Figure 8, (**b**) is composed of eight images in Figure 9, (**c**) is composed of eight images in Figure 10, (**d**) is composed of eight images in Figure 11.

**Figure 15:**
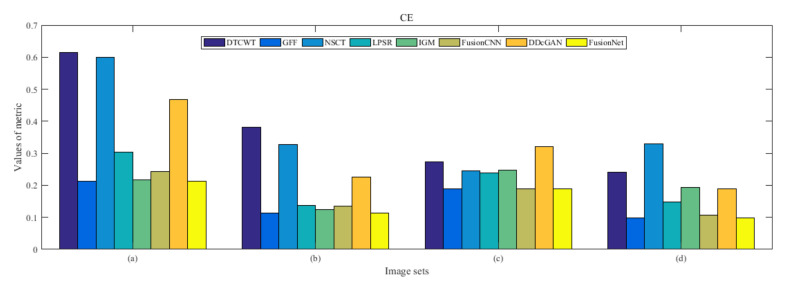
Values of cross entropy (CE) in the fused images (MRI-SPECT): (**a**) is composed of eight images in Figure 8, (**b**) is composed of eight images in Figure 9, (**c**) is composed of eight images in Figure 10, (**d**) is composed of eight images in Figure 11.

**Figure 16:**
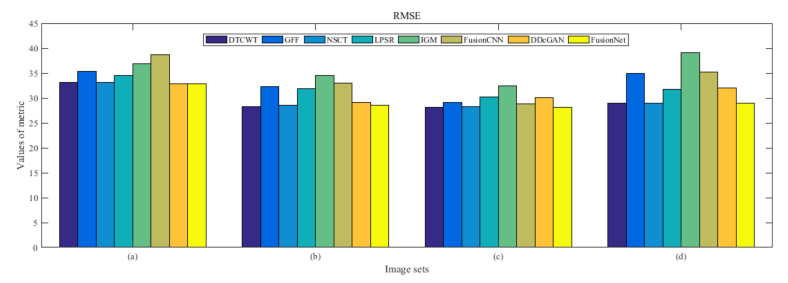
Values of root mean squared error (RMSE) in the fused images (MRI-SPECT): (**a**) is composed of eight images in Figure 8, (**b**) is composed of eight images in Figure 9, (**c**) is composed of eight images in Figure 10, (**d**) is composed of eight images in Figure 11.

**Figure 17:**
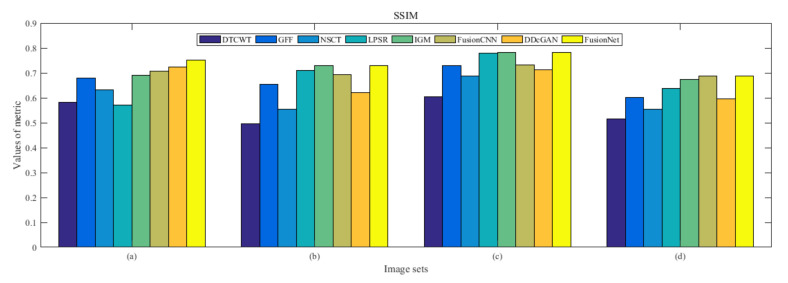
Values of structural similarity (SSIM) in the fused images (MRI-SPECT): (**a**) is composed of eight images in Figure 8, (**b**) is composed of eight images in Figure 9, (**c**) is composed of eight images in Figure 10, (**d**) is composed of eight images in Figure 11.

**Figure 18:**
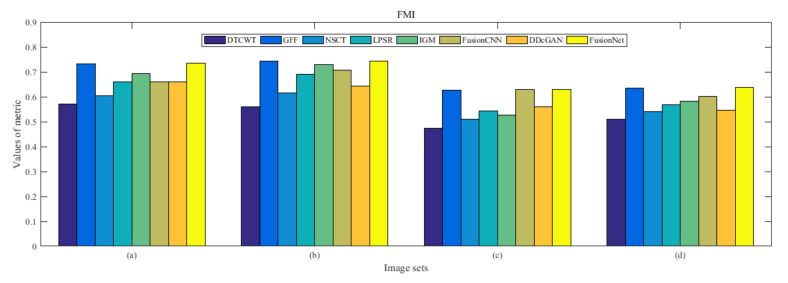
Values of feature mutual information (FMI) in the fused images (MRI-SPECT): (**a**) is composed of eight images in Figure 8, (**b**) is composed of eight images in Figure 9, (**c**) is composed of eight images in Figure 10, (**d**) is composed of eight images in Figure 11.

**Figure 19:**
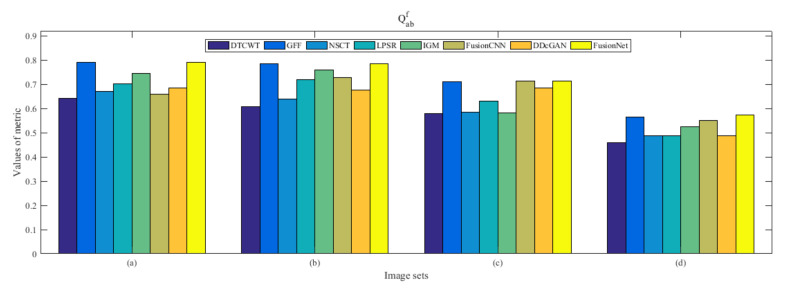
Values of $Q_{ab}^{f}$ in the fused images (MRI-SPECT): (**a**) is composed of eight images in Figure 8, (**b**) is composed of eight images in Figure 9, (**c**) is composed of eight images in Figure 10, (**d**) is composed of eight images in Figure 11.

**Figure 20:**
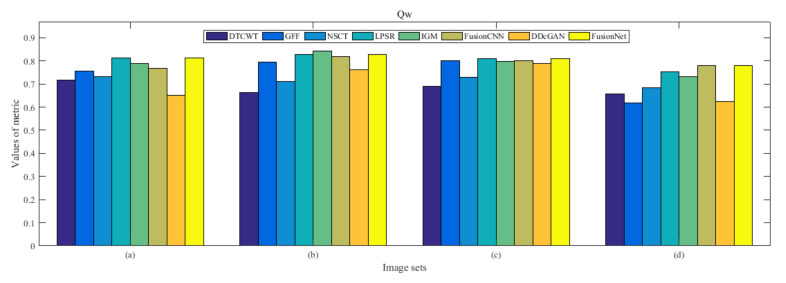
Values of $Q_{w}$ in the fused images (MRI-SPECT): (**a**) is composed of eight images in Figure 8, (**b**) is composed of eight images in Figure 9, (**c**) is composed of eight images in Figure 10, (**d**) is composed of eight images in Figure 11.

**Figure 21:**
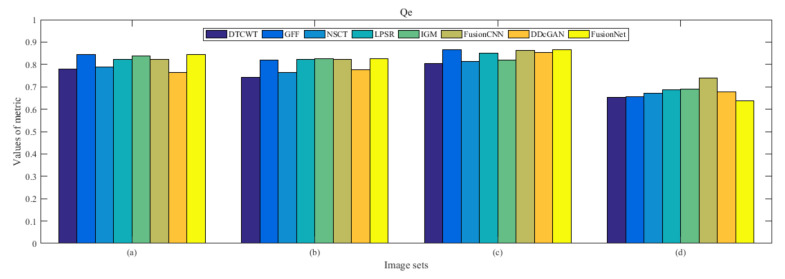
Values of $Q_{e}$ in the fused images (MRI-SPECT): (**a**) is composed of eight images in Figure 8, (**b**) is composed of eight images in Figure 9, (**c**) is composed of eight images in Figure 10, (**d**) is composed of eight images in Figure 11.

**Figure 22:**
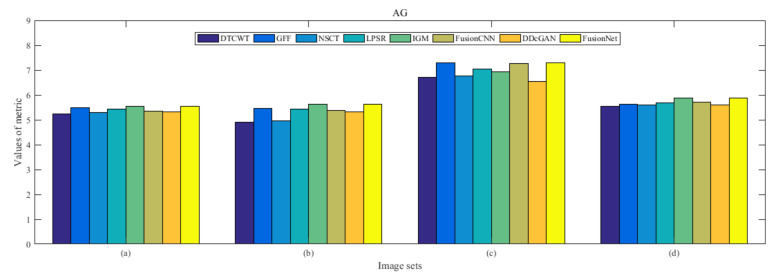
Values of average gradient (AG) in the fused images (MRI-SPECT): (**a**) is composed of eight images in Figure 8, (**b**) is composed of eight images in Figure 9, (**c**) is composed of eight images in Figure 10, (**d**) is composed of eight images in Figure 11.

**Figure 23:**
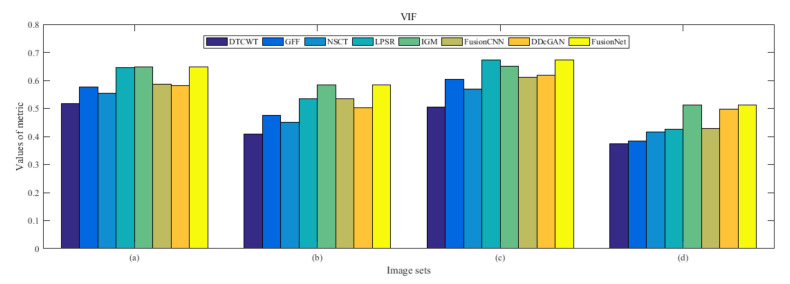
Values of visual information fidelity (VIF) in the fused images (MRI-SPECT): (**a**) is composed of eight images in Figure 8, (**b**) is composed of eight images in Figure 9, (**c**) is composed of eight images in Figure 10, (**d**) is composed of eight images in Figure 11.

From Figure 12 and Figure 13, we find that algorithm obtains the greatest preference, indicating that FusionNet can get better fusion results from the subjective aspect. The objective evaluation metrics of fused images of all methods in the MRI-SPECT image fusion are shown in Figure 14, Figure 15, Figure 16, Figure 17, Figure 18, Figure 19, Figure 20, Figure 21, Figure 22 and Figure 23. Our FusionNet performs well on *SSIM*, $Q_{ab}^{f}$, $Q_{w}$, and *VIF* in MRI and SPECT fusion images. In terms of *EN*, *CE*, *AG*, and *FMI*, our algorithm is slightly inferior to DDcGAN, IGM, GFF, and FusionCNN. As for the remaining two indicators, our algorithm is similar to other algorithms. Subjective evaluation and objective evaluation are inconsistent sometimes; however, in medical diagnosis, objective evaluation cannot be a complete basis for diagnosis, while subjective evaluation is often more comprehensive. However, the fusion images got by FusionNet have achieved good results in subjective and objective evaluation.

### 4.3. The Fusion of MRI-FDG

Fludeoxyglucose (FDG) image in cancer diagnosis plays an important role; at the same time, it provides the functional information that can predict a pathological reaction to certain types of cancer treatment. As a kind of PET image, FDG image has some features of PET image, such as texture analysis [54] and shape analysis [55], may also provide additional knowledge associated with the treatment outcome. However, FDG image has no structural information, which is its biggest defect. Therefore, the fusion of MRI and FDG can give doctors a great help in the process of cancer diagnosis.

**Figure 24:**
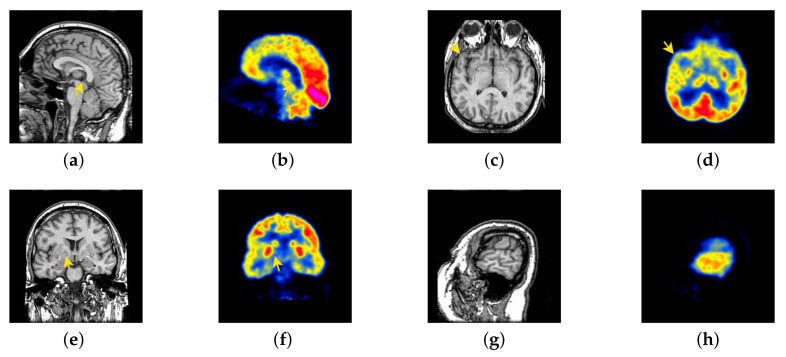
Four pairs of MRI-Fludeoxyglucose (FDG) source images: (**a**,**c**,**e**,**g**) are MRI images; (**b**,**d**,**f**,**h**) are FDG images.

**Figure 25:**
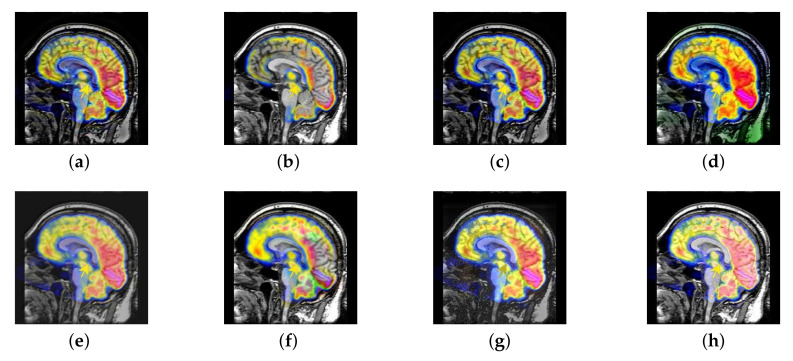
Fused medical images obtained by different algorithms (Figure 24a,b): (**a**) DTCWT, (**b**) GFF, (**c**) NSCT, (**d**) LPSR, (**e**) IGM, (**f**) FusionCNN, (**g**) DDcGAN, and (**h**) FusionNet.

**Figure 26:**
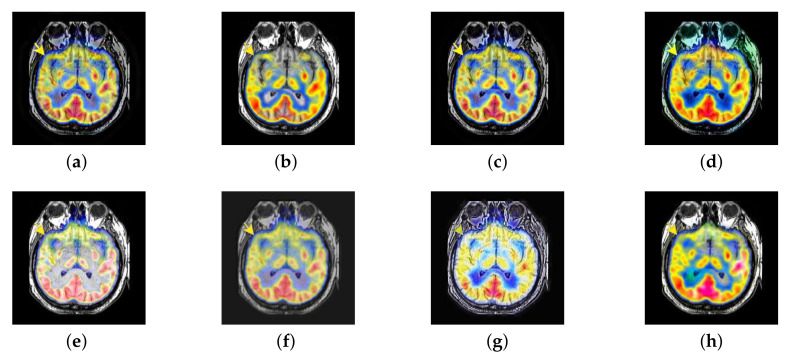
Fused medical images obtained by different algorithms (Figure 24c,d): (**a**) DTCWT, (**b**) GFF, (**c**) NSCT, (**d**) LPSR, (**e**) IGM, (**f**) FusionCNN, (**g**) DDcGAN, and (**h**) FusionNet.

**Figure 27:**
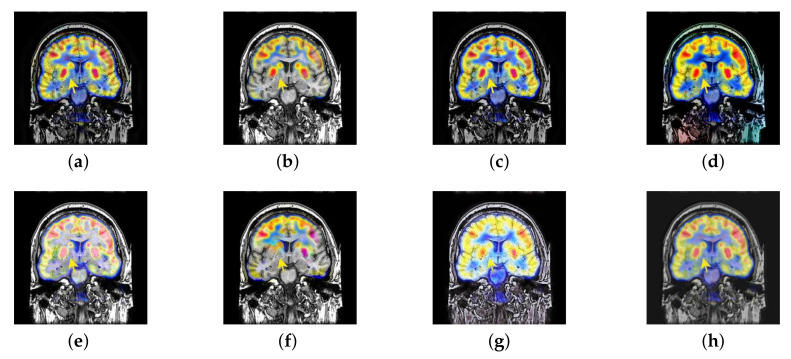
Fused medical images obtained by different algorithms (Figure 24e,f): (**a**) DTCWT, (**b**) GFF, (**c**) NSCT, (**d**) LPSR, (**e**) IGM, (**f**) FusionCNN, (**g**) DDcGAN, and (**h**) FusionNet.

**Figure 28:**
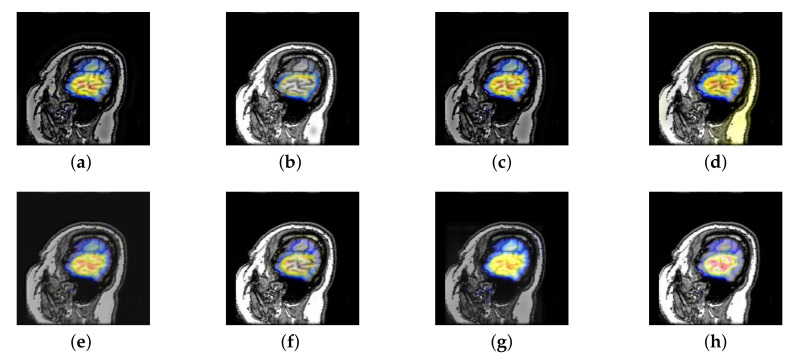
Fused medical images obtained by different algorithms (Figure 24g,h): (**a**) DTCWT, (**b**) GFF, (**c**) NSCT, (**d**) LPSR, (**e**) IGM, (**f**) FusionCNN, (**g**) DDcGAN, and (**h**) FusionNet.

In this section, all FDG images are derived from the normal human brain, but the angle is chosen differently in Figure 24. In Figure 25, Figure 26, Figure 27 and Figure 28, we find that the color information obtained by the image fusion method based on NSCT, DTCWT, and LPSR is better preserved, but the structure information is lost more. The fusion image based on GFF, IGM, and FusionCNN method retains the complete structure information in the MRI image, but the color obtained from the FDG image is distorted. The image color information obtained by DDcGAN fusion method is too bright, resulting in unclear color area details and low contrast of color region. In contrast, the image obtained by our algorithm has moderate brightness of color information, complete structure information, and complete biological detail information.

**Figure 29:**
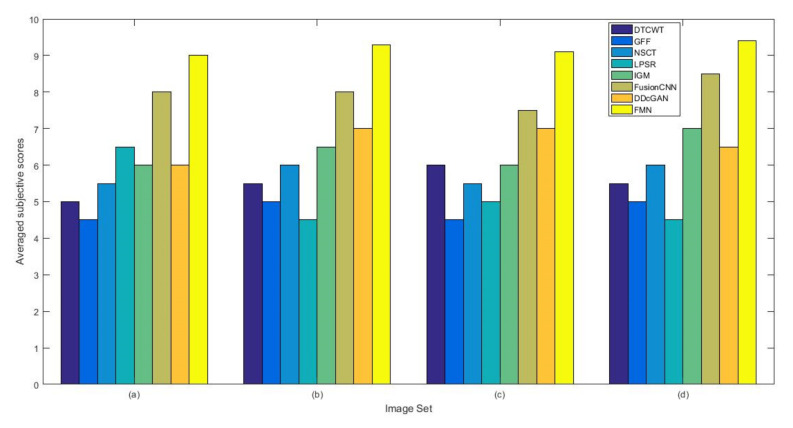
The averaged subjective scores of the fused images (MRI-FDG): (**a**) is composed of eight images in Figure 25, (**b**) is composed of eight images in Figure 26, (**c**) is composed of eight images in Figure 27, (**d**) is composed of eight images in Figure 28.

**Figure 30:**
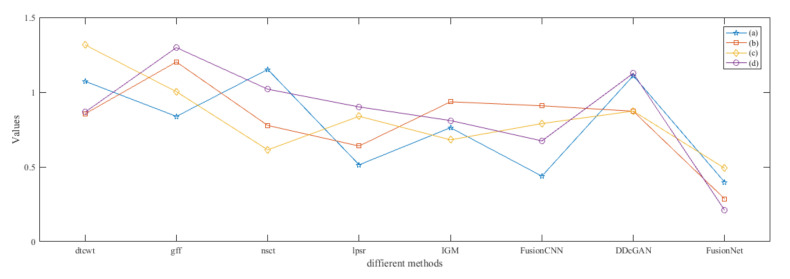
The standard deviations of subjective scores of the fused images (MRI-SPECT).

**Figure 31:**
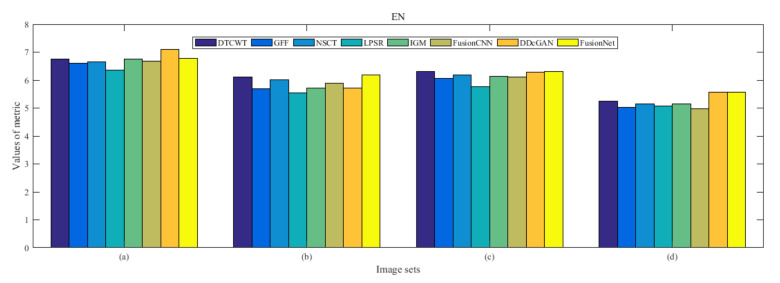
Values of entropy (EN) in the fused images (MRI-FDG): (**a**) is composed of eight images in Figure 25, (**b**) is composed of eight images in Figure 26, (**c**) is composed of eight images in Figure 27, (**d**) is composed of eight images in Figure 28.

**Figure 32:**
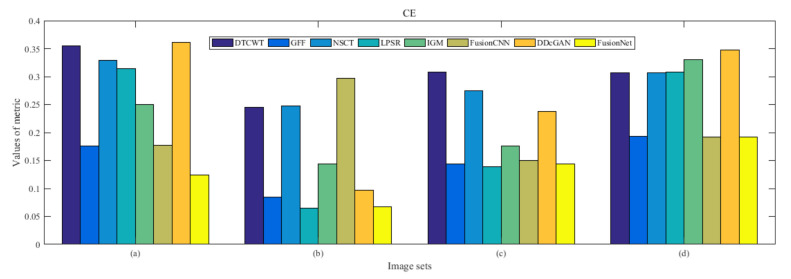
Values of cross entropy (CE) in the fused images (MRI-FDG): (a) is composed of eight images in Figure 25, (b) is composed of eight images in Figure 26, (c) is composed of eight images in Figure 27, (d) is composed of eight images in Figure 28.

**Figure 33:**
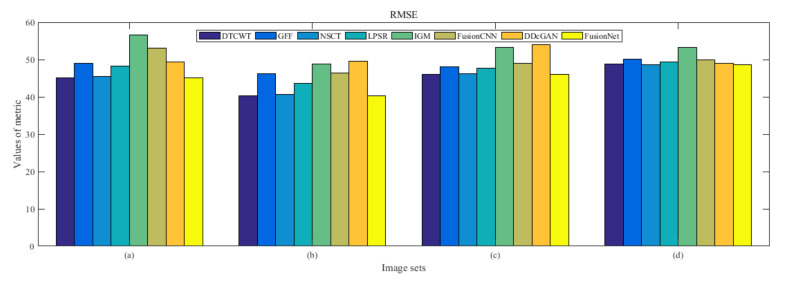
Values of root mean squared error (RMSE) in the fused images (MRI-FDG): (**a**) is composed of eight images in Figure 25, (**b**) is composed of eight images in Figure 26, (**c**) is composed of eight images in Figure 27, (**d**) is composed of eight images in Figure 28.

**Figure 34:**
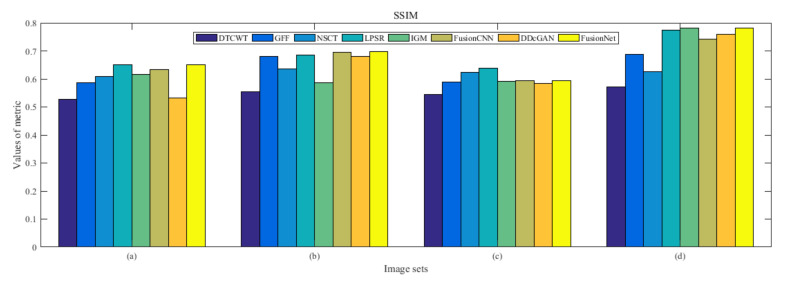
Values of structural similarity (SSIM) in the fused images (MRI-FDG): (**a**) is composed of eight images in Figure 25, (**b**) is composed of eight images in Figure 26, (**c**) is composed of eight images in Figure 27, (**d**) is composed of eight images in Figure 28.

**Figure 35:**
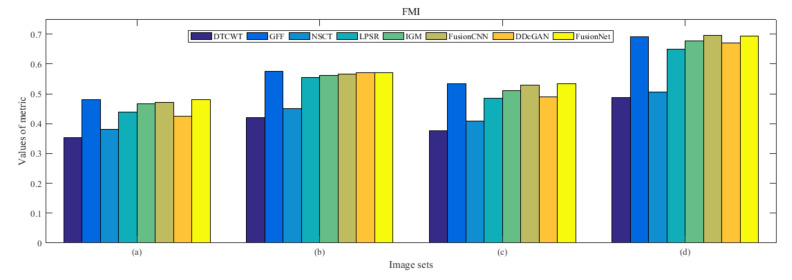
Values of feature mutual information (FMI) in the fused images (MRI-FDG): (**a**) is composed of eight images in Figure 25, (**b**) is composed of eight images in Figure 26, (**c**) is composed of eight images in Figure 27, (**d**) is composed of eight images in Figure 28.

**Figure 36:**
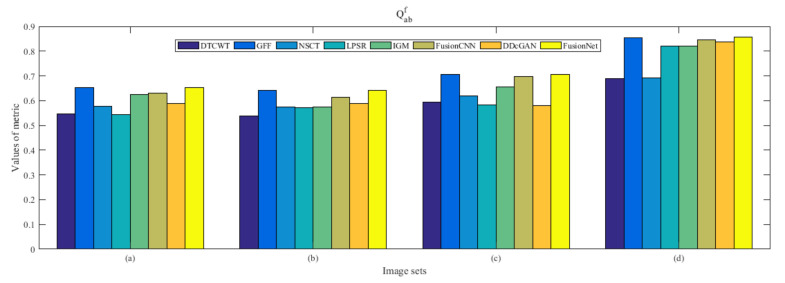
Values of $Q_{ab}^{f}$ in the fused images (MRI-FDG): (**a**) is composed of eight images in Figure 25, (**b**) is composed of eight images in Figure 26, (**c**) is composed of eight images in Figure 27, (**d**) is composed of eight images in Figure 28.

**Figure 37:**
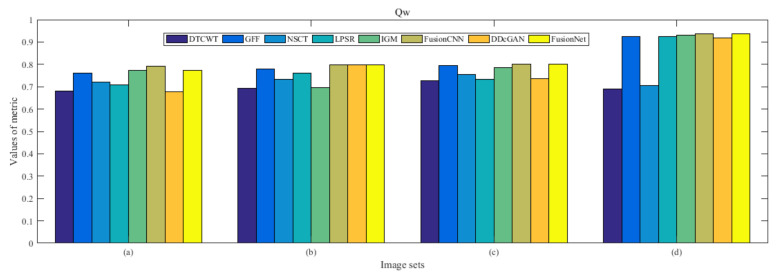
Values of $Q_{w}$ in the fused images (MRI-FDG): (**a**) is composed of eight images in Figure 25, (**b**) is composed of eight images in Figure 26, (**c**) is composed of eight images in Figure 27, (**d**) is composed of eight images in Figure 28.

**Figure 38:**
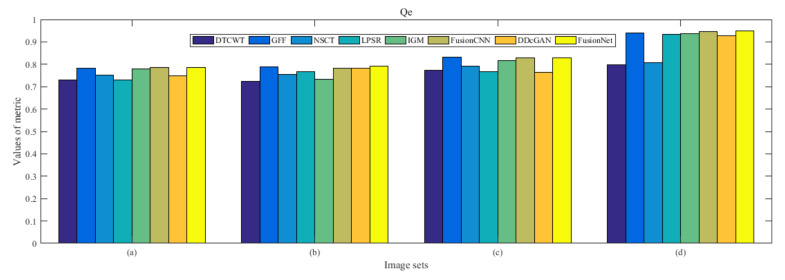
Values of $Q_{e}$ in the fused images (MRI-FDG): (**a**) is composed of eight images in Figure 25, (**b**) is composed of eight images in Figure 26, (**c**) is composed of eight images in Figure 27, (**d**) is composed of eight images in Figure 28.

**Figure 39:**
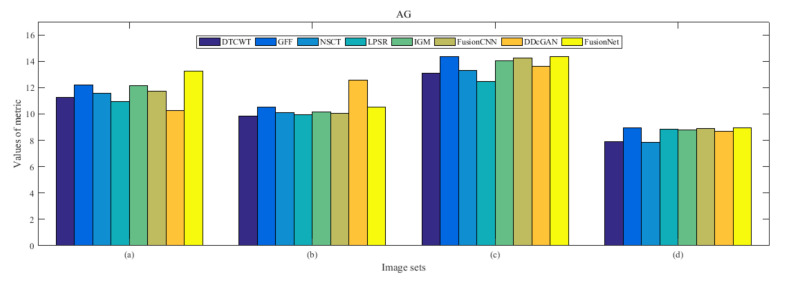
Values of average gradient (AG) in the fused images (MRI-FDG): (**a**) is composed of eight images in Figure 25, (**b**) is composed of eight images in Figure 26, (**c**) is composed of eight images in Figure 27, (**d**) is composed of eight images in Figure 28.

**Figure 40:**
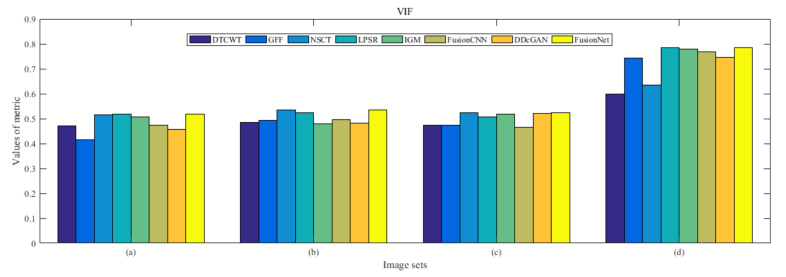
Values of visual information fidelity (VIF) in the fused images (MRI-FDG): (**a**) is composed of eight images in Figure 25, (**b**) is composed of eight images in Figure 26, (**c**) is composed of eight images in Figure 27, (**d**) is composed of eight images in Figure 28.

The averaged subjective scores of MRI-FDG fused images obtained by 8 algorithms are shown in Figure 29 and Figure 30, and objective evaluation indicators are all shown from Figure 31, Figure 32, Figure 33, Figure 34, Figure 35, Figure 36, Figure 37, Figure 38, Figure 39 and Figure 40. Overall, our algorithm performs well in *EN*, *FMI*, *RMSE*, *AG*, *SSIM*, $Q_{w}$, $Q_{e}$, and $Q_{ab}^{f}$. Our algorithm is the best of eight algorithms in the fusion of image structure information. In the metric of cross entropy and visual information fidelity, our algorithm is slightly inferior to other algorithms. However, from the overall evaluation, the algorithm has obvious advantages in fusion MRI and FDG.

### 4.4. The Fusion of MRI-CBF

Cerebral blood flow diagram (CBF), which indicates the amount of blood flow in brain tissue with color. Red, yellow, green, blue, and black successively indicate the amount of blood flow from more to less. It is mainly used to detect the blood flow supply condition, elasticity, tension, and peripheral resistance. However, with the development of medical science, CBF image is often inferior in the diagnosis of brain diseases due to its lack of structural information. Therefore, MRI which is good at expressing structural information, is introduced to fuse in the current trend of brain medicine.

**Figure 41:**
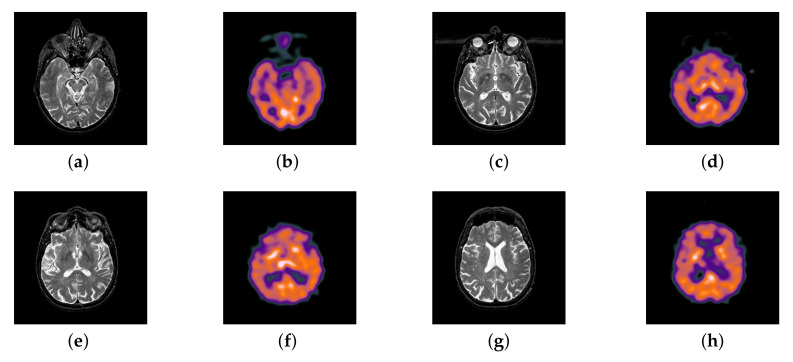
Four pairs of MRI-cerebral blood flow diagram (CBF) source images: (**a**,**c**,**e**,**g**) are MRI images; (**b**,**d**,**f**,**h**) are CBF images.

**Figure 42:**
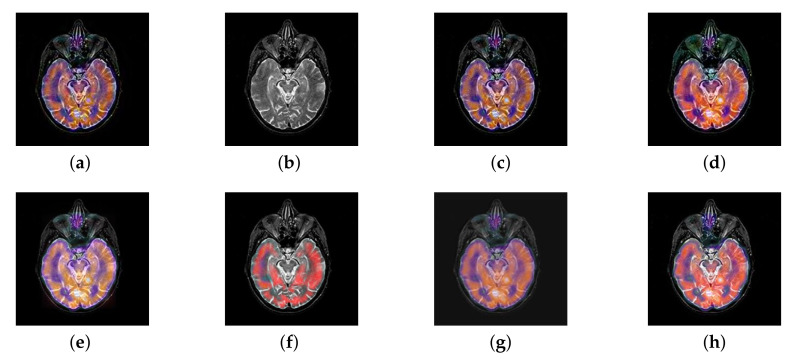
Fused medical images obtained by different algorithms (Figure 41a,b): (**a**) DTCWT, (**b**) GFF, (**c**) NSCT, (**d**) LPSR, (**e**) IGM, (**f**) FusionCNN, (**g**) DDcGAN, and (**h**) FusionNet.

**Figure 43:**
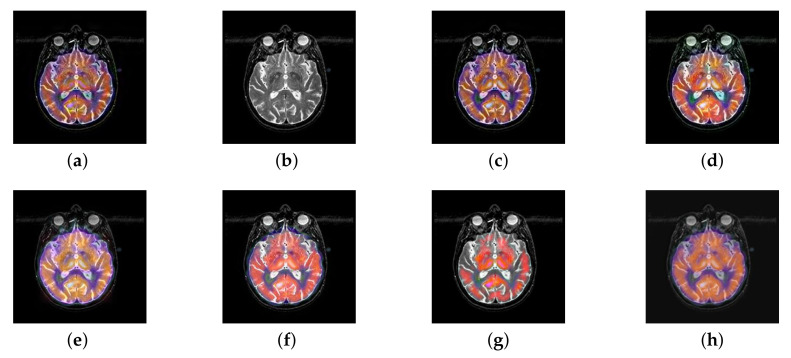
Fused medical images obtained by different algorithms (Figure 41c,d): (**a**) DTCWT, (**b**) GFF, (**c**) NSCT, (**d**) LPSR, (**e**) IGM, (**f**) FusionCNN, (**g**) DDcGAN, and (**h**) FusionNet.

**Figure 44:**
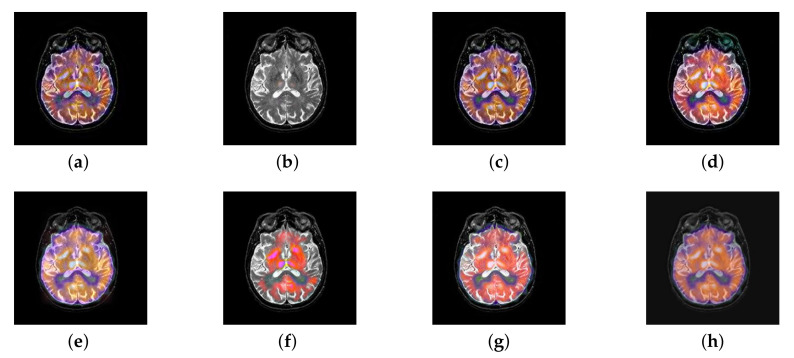
Fused medical images obtained by different algorithms (Figure 41e,f): (**a**) DTCWT, (**b**) GFF, (**c**) NSCT, (**d**) LPSR, (**e**) IGM, (**f**) FusionCNN, (**g**) DDcGAN, and (**h**) FusionNet.

**Figure 45:**
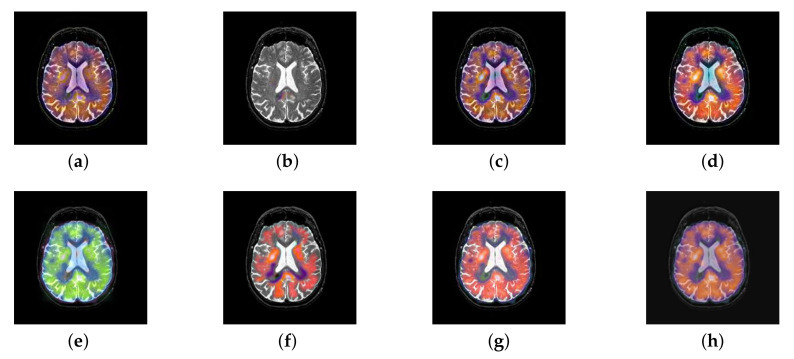
Fused medical images obtained by different algorithms (Figure 41g,h): (**a**) DTCWT, (**b**) GFF, (**c**) NSCT, (**d**) LPSR, (**e**) IGM, (**f**) FusionCNN, (**g**) DDcGAN, and (**h**) FusionNet.

In this section, there are four image sets to fuse, each containing a MRI image and its corresponding CBF image in Figure 41. In Figure 42, Figure 43, Figure 44 and Figure 45, it can be seen that the structural information of fusion images obtained by our algorithm is complete. The color is not distorted, and the spectral features are natural. The fusion image based on DTCWT and NSCT algorithm have high color fidelity but less structural information. Other algorithms, such as IGM, LPSR, and FusionCNN, only focus on the structural information of the MRI image and ignore the color information of the fused image. Although the image structure information obtained by DDcGAN fusion algorithm is relatively complete, the edge of color information is not clear, which has a great influence on image contrast.

**Figure 46:**
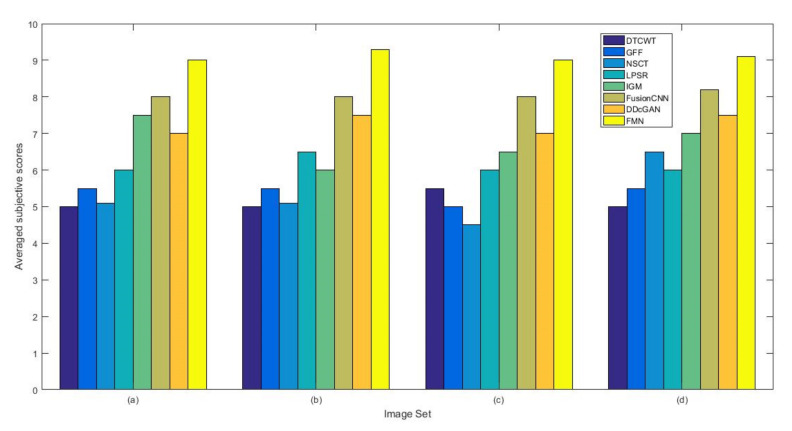
The averaged subjective scores of the fused images (MRI-CBF): (**a**) is composed of eight images in Figure 42, (**b**) is composed of eight images in Figure 43, (**c**) is composed of eight images in Figure 44, (**d**) is composed of eight images in Figure 45.

**Figure 47:**
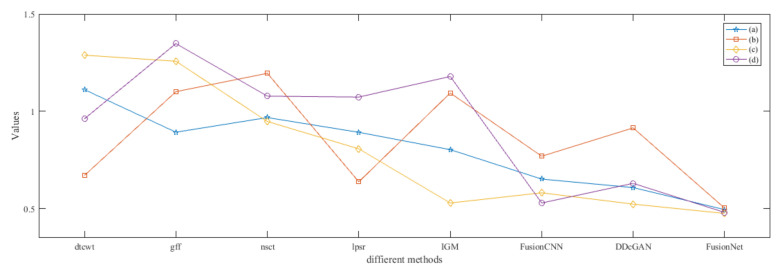
The standard deviations of subjective scores of the fused images (MRI-SPECT).

**Figure 48:**
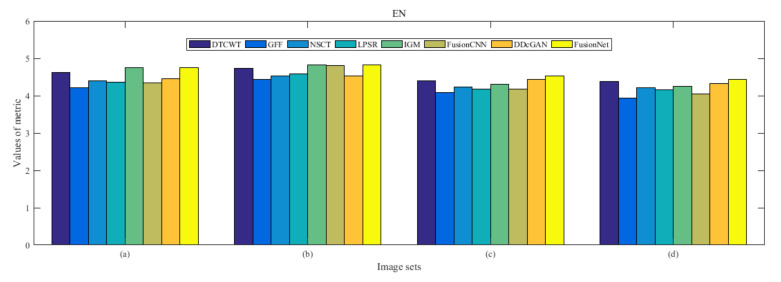
Values of entropy (EN) in the fused images (MRI-CBF): (**a**) is composed of eight images in Figure 42, (**b**) is composed of eight images in Figure 43, (**c**) is composed of eight images in Figure 44, (**d**) is composed of eight images in Figure 45.

**Figure 49:**
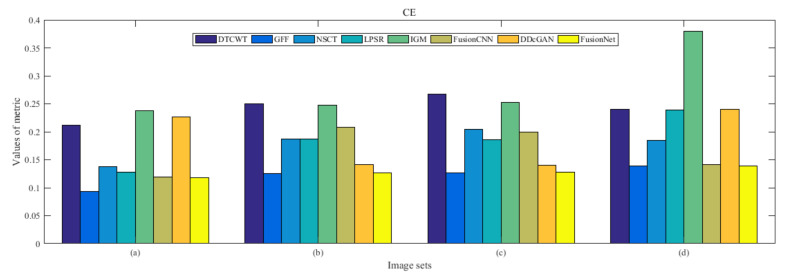
Values of cross entropy (CE) in the fused images (MRI-CBF): (**a**) is composed of eight images in Figure 42, (**b**) is composed of eight images in Figure 43, (**c**) is composed of eight images in Figure 44, (**d**) is composed of eight images in Figure 45.

**Figure 50:**
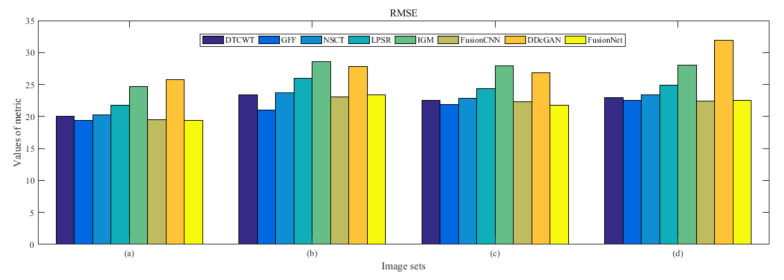
Values of root mean squared error (RMSE) in the fused images (MRI-CBF): (**a**) is composed of eight images in Figure 42, (**b**) is composed of eight images in Figure 43, (**c**) is composed of eight images in Figure 44, (**d**) is composed of eight images in Figure 45.

**Figure 51:**
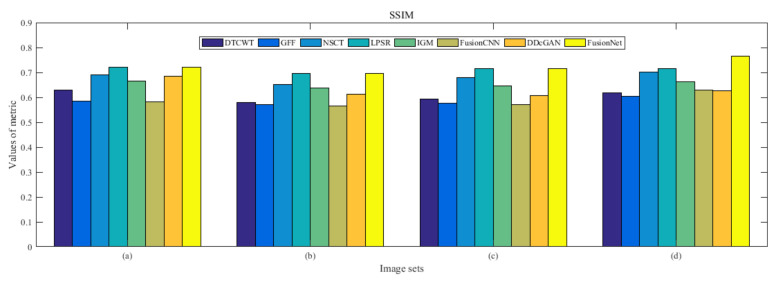
Values of structural similarity (SSIM) in the fused images (MRI-CBF): (**a**) is composed of eight images in Figure 42, (**b**) is composed of eight images in Figure 43, (**c**) is composed of eight images in Figure 44, (**d**) is composed of eight images in Figure 45.

**Figure 52:**
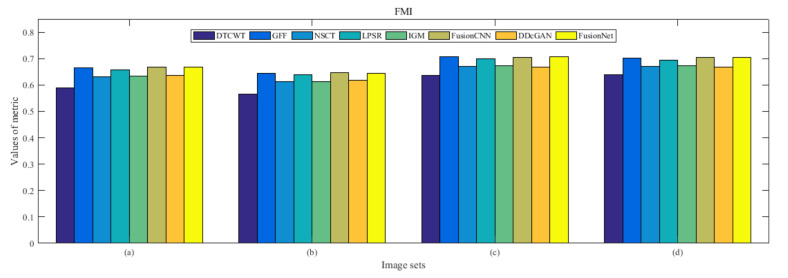
Values of feature mutual information (FMI) in the fused images (MRI-CBF): (**a**) is composed of eight images in Figure 42, (**b**) is composed of eight images in Figure 43, (**c**) is composed of eight images in Figure 44, (**d**) is composed of eight images in Figure 45.

**Figure 53:**
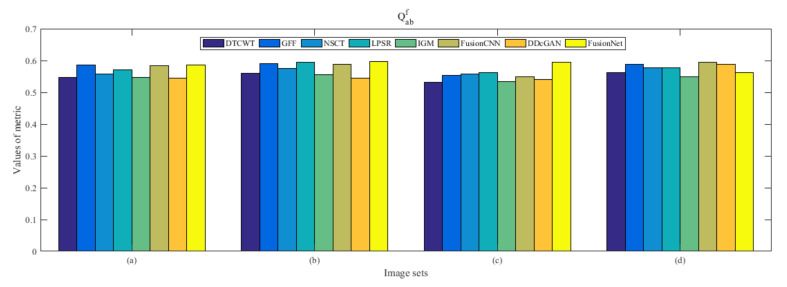
Values of $Q_{ab}^{f}$ in the fused images (MRI-CBF): (**a**) is composed of eight images in Figure 42, (**b**) is composed of eight images in Figure 43, (**c**) is composed of eight images in Figure 44, (**d**) is composed of eight images in Figure 45.

**Figure 54:**
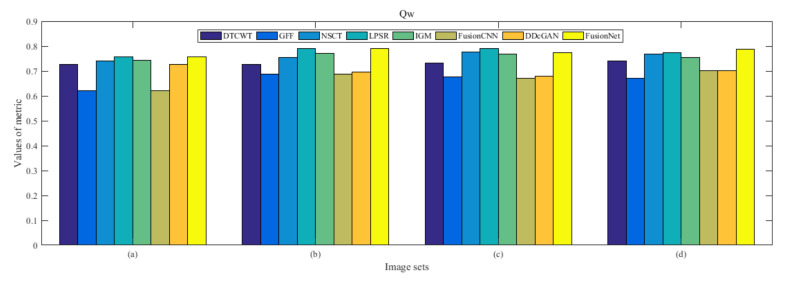
Values of $Q_{w}$ in the fused images (MRI-CBF): (**a**) is composed of eight images in Figure 42, (**b**) is composed of eight images in Figure 43, (**c**) is composed of eight images in Figure 44, (**d**) is composed of eight images in Figure 45.

**Figure 55:**
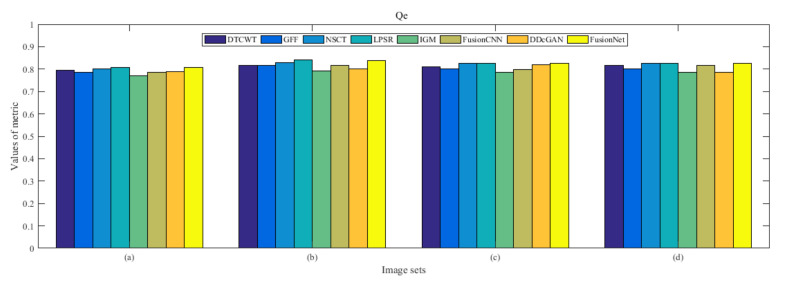
Values of $Q_{e}$ in the fused images (MRI-CBF): (**a**) is composed of eight images in Figure 42, (**b**) is composed of eight images in Figure 43, (**c**) is composed of eight images in Figure 44, (**d**) is composed of eight images in Figure 45.

**Figure 56:**
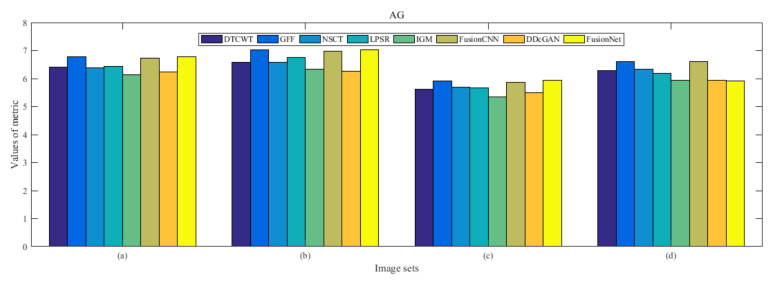
Values of average gradient (AG) in the fused images (MRI-CBF): (**a**) is composed of eight images in Figure 42, (**b**) is composed of eight images in Figure 43, (**c**) is composed of eight images in Figure 44, (**d**) is composed of eight images in Figure 45.

**Figure 57:**
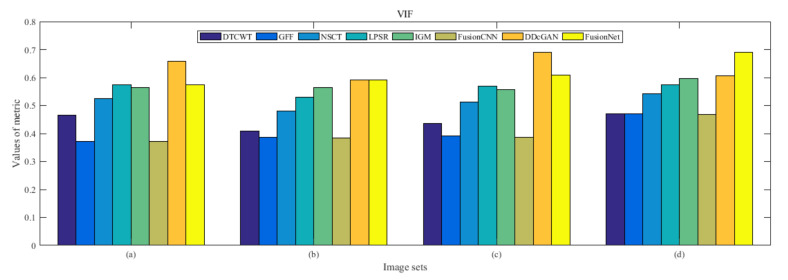
Values of visual information fidelity (VIF) in the fused images (MRI-CBF): (**a**) is composed of eight images in Figure 42, (**b**) is composed of eight images in Figure 43, (**c**) is composed of eight images in Figure 44, (**d**) is composed of eight images in Figure 45.

The averaged subjective scores of MRI-CBF fusion images obtained by the above methods are shown from Figure 46 and Figure 47. Our fusion algorithm has obvious advantages in *EN*, *FMI*, *SSIM*, $Q_{w}$, $Q_{ab}^{f}$, and *AG* from Figure 48, Figure 49, Figure 50, Figure 51, Figure 52, Figure 53, Figure 54, Figure 55, Figure 56 and Figure 57. Other metrics are inferior to those of FusionCNN, GFF, and LPSR fusion algorithm. However, as we have mentioned before, there may be inconsistency between subjective indicators and objective indicators, but this does not affect the assessment of image quality.

### 4.5. Metrics Discussion

What we do is the image fusion of structured images and multi-type functional images. Due to the diversity of functional image categories and their different imaging principles, the ten indicators for objective evaluation cannot all be equally good. But the reason why we list all ten indicators is to allow all multi-modal medical image fusions to be evaluated fairly under the same quality evaluation system, and the other is to distinguish which indicators are more suitable for which type of image fusion evaluation. In MRI-SPECT fusion, our fusion results are slightly worse than some MST image fusion algorithms on *RMSE*. The overall characteristic of MST fusion algorithm is that the loss of image information is small and fast. But it cannot handle texture and details well, resulting in unclear texture and blurry details of the fused image. So, it performs well in RMSE, But the fusion effect is not satisfactory. On the indicator of *Qe*, our model is inferior to GFF, IGM, and FusionCNN on several pictures. The difference is extremely small, all of which are four decimal places. This can only show that the above three algorithms are slightly better than our model in terms of edge similarity structure. But in the final fusion image, we can also clearly see that their colors are poorly fused, either there is almost no color, or the color distortion is particularly severe. Therefore, *RMSE* and *Qe* are not the most important evaluation indicators in MRI-SPECT. In MRI-FDG fusion, our model is weaker than LPSR in both *CE* and *VIF* performance. The LPSR algorithm is an image fusion algorithm that completely relies on the training dictionary. The more complete the dictionary, the more information can be obtained in the fused image, but this does not mean that the fused image will have a better effect, and it will be more helpful to the doctor. The results of the experiment just verify my point of view. The image obtained by the LPSR algorithm has high visual fidelity, and the fused image contains a lot of information of the source image, but the same location information is too much and blurred, which is not what doctors want. Therefore, *CE* and *VIF* are not the most important indicators in MRI-FDG fusion. In MRI-CBF fusion, in the above four indicators, the performance of our model is not as good as GFF, LPSR, and DDcGAN, respectively. The GFF and LPSR algorithms have been explained in detail above and will not be repeated here. The DDcGAN algorithm is an improvement of the GAN algorithm and is better than our algorithm on *VIF*. Because its fusion process is a game process, the output fusion image has high brightness and rich color information, resulting in lack of structural details, which affects observation. In summary, *CE*, *LPSR*, *Qe*, and *VIF* can be used as reference evaluation metrics in our model, but they are not the most important evaluation metrics.

### 4.6. Proposed Framework Analysis

**Figure 58:**
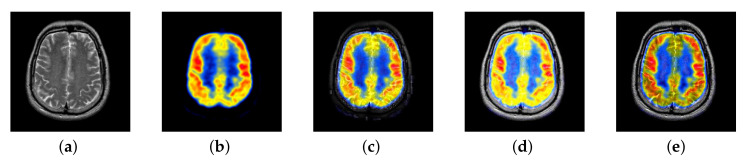
Source images and fused medical images obtained by different algorithms: (**a**) MRI, (**b**) positron emission tomography (PET), (**c**) DenseNet, (**d**) traditional intuitive fuzzy processing (IFP), and (**e**) FusionNet.

Our proposed FusionNet is inspired by DenseNet. DenseNet has achieved great success in infrared and visible image fusion, however, there are great differences between medical images and two types of images that are mentioned above. DenseNet directly does multi-mode medical image fusion, which is not ideal. Therefore, we have done many improvements. In view of the advantages of intuitionistic fuzzy sets in image processing, we improved the intuitionistic fuzzy sets as part of image preprocessing and added them to our framework. In our experiment, two methods, DenseNet and traditional IFP, were introduced to help us analyze our own methods. Figure 58a,b are source images; Figure 58c is the result of DenseNet fusion; Figure 58d is the fusion result of traditional IFP; Figure 58e is the fusion result of our proposed method.

In DenseNet, all middle layer reuses many features of the image, resulting there are few information features, low image brightness, and loss of edge structure information, thus losing the significance of MRI fusion. In traditional IFP, the membership image, the non-membership image and the hesitation image are obtained by the membership function of the multi-mode medical image. Then, the membership image is taken for subsequent fusion operations. This approach allows us to remove more useful information, such as textures of structural images. Considering the disadvantages of DenseNet and traditional IFP, we try to use only the second layer as the unique feature reuse layer to ensure that the respective features of the source image, which can be perfectly reflected in the final fused image. At the same time, the traditional IFP is improved to improve its ability to retain valid information. As can be seen from the experimental results of the following images, FusionNet can retain the structural features and color information required for medical diagnosis in the fused images.

### 4.7. Computational Time Comparison

**Figure 59:**
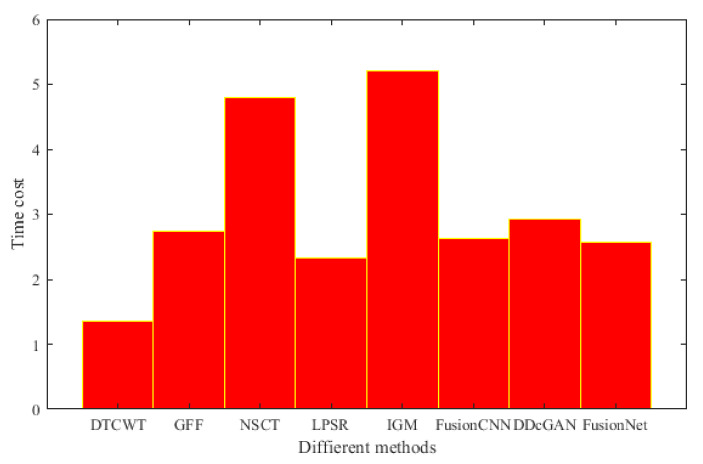
The time complexity of different types of multi-modal medical images.

The time complexity of our method is compared with that of other fusion techniques. In Figure 59, we listed the running time of different fusion methods in the fusion of two 256×256 pixel multi-modal medical images under the condition of 2.20 Ghz CPU and 16GB RAM. Among them, DTCWT, GFF, NSCT, and IGM are implemented in pure MATLAB, while LPSR, FusionCNN, DDcGAN, and the method in this paper adopt MATLAB and Python mixed programming. We can see that our method has a lower computational efficiency compared with the above comparison method.

As can be seen from Figure 59, the time complexity of NSCT-based algorithm is relatively high, and the fusion time is generally more than 4 s. The algorithm based on DTCWT has the lowest time complexity and fusion time is less than 2 s. The fusion results obtained by the two methods are similar, the color information is complete, but the edge information is not ideal. IGM algorithm has the highest time complexity, but the image after fusion is too bright, so the details are not clear. The time complexity of the remaining algorithms is similar to that of FusionNet; however, their fusion results are not as good as that of FusionNet.

## 5. Conclusions and Future Development

In this article, we propose a multi-modal medical image fusion model based on feature multiplexing. Compared with other models, it has four main advantages: (1) Our model is the first model that is close to the application of multi-modal medical image fusion, that is, subjective evaluation is completely dependent on the prior knowledge of imaging, rather than simply relying on personal preference. (2) Our model uses an appropriate feature reuse layer instead of a complex DenseNet for feature extraction, which not only increases the utilization of features in the last layer but also reduces the time complexity. (3) Since our experimental data is not very limited, especially in functional images, three categories have been involved, and the diversity of image data has been realized, so that the robustness of our model has been greatly improved. (4) For the first time, the cross-entropy and structural similarity joint loss function is introduced into the image fusion model to optimize the model, which promotes the model to reconstruct images with more detailed texture and color. The model has good performance in all categories of objective indicators, especially on SSIM, EN, Qabf, FMI, Qw, and AG. Although the performance on RMSE, Qe, CE, and VIF was average, it did not affect the final fusion effect. Diversified experimental data and comprehensive evaluation methods once again prove the stability of our model in multi-modal medical image fusion. At the same time, it has abandoned the previous concept that only medical images were used as the object of image fusion, making it lose its application significance.

This work has laid a pioneering foundation for image fusion applications of convolutional neural networks in the real medical field. However, despite the extensive experimental results verifying the advantages of the proposed model, there are still some problems that need to be further resolved in order to obtain a better performance image fusion model. First of all, our selection of structural images is currently a bit single. All we select are structural images in MRI. There are actually many types of medical structural images, such as CT, X-ray imaging, etc., so the data set is expanded to a wider range Structured data sets may improve the performance of the model. Secondly, the multi-modal medical image is preprocessed before entering our model, and the image is enhanced using the intuitive fuzzy set. Can we directly integrate a multi-modal image enhancement algorithm based on the prior knowledge of imaging in our encoder? This will have more application significance. Thirdly, our source images are always registered images, but, in actual operation, it is difficult to obtain registered images. Therefore, the development of image fusion models for non-registered images has great potential. Finally, our model can already obtain the multi-modal fusion image that doctors need, but whether the fusion details of the image can be used to discover the causes of abnormalities in the tissue will be challenging and of far-reaching significance.
